# Genetically Engineered Microorganisms and Their Impact on Human Health

**DOI:** 10.1155/2024/6638269

**Published:** 2024-03-09

**Authors:** Marzie Mahdizade Ari, Leila Dadgar, Zahra Elahi, Roya Ghanavati, Behrouz Taheri

**Affiliations:** ^1^Department of Microbiology, School of Medicine, Iran University of Medical Sciences, Tehran, Iran; ^2^Microbial Biotechnology Research Centre, Iran University of Medical Sciences, Tehran, Iran; ^3^Behbahan Faculty of Medical Sciences, Behbahan, Iran; ^4^Department of Biotechnology, School of Medicine, Ahvaz Jundishapour University of medical Sciences, Ahvaz, Iran

## Abstract

The emergence of antibiotic-resistant strains, the decreased effectiveness of conventional therapies, and the side effects have led researchers to seek a safer, more cost-effective, patient-friendly, and effective method that does not develop antibiotic resistance. With progress in synthetic biology and genetic engineering, genetically engineered microorganisms effective in treatment, prophylaxis, drug delivery, and diagnosis have been developed. The present study reviews the types of genetically engineered bacteria and phages, their impacts on diseases, cancer, and metabolic and inflammatory disorders, the biosynthesis of these modified strains, the route of administration, and their effects on the environment. We conclude that genetically engineered microorganisms can be considered promising candidates for adjunctive treatment of diseases and cancers.

## 1. Introduction

Since thousands of years ago, humans have turned to make changes in the characteristics of animals, plants, and microbes. The result of the changes is the creation of modified strains used in the food industry. The acquired success engaged researchers to develop more diverse genetic engineering techniques [1]. Genetic engineering, also known as genetic modification, is the process of using laboratory tools to change the nucleic acid sequence of an organism by removing or adding base pairs, inserting or inactivating an unnecessary virulence gene by creating a new characteristic in genetically engineered microorganisms (GEMs). Formerly, humans made changes in microbes to produce foods such as bread and wine, while nowadays, genetic engineering of microbes has been used for industry and clinical applications. Among microbes, yeast and bacteria, lactic acid bacteria (LAB), and *Saccharomyces cerevisiae* are most organisms undergoing chemical changes [2]. As genome sequencing and genetic techniques are developed and become powerful genomic tools, it is possible to make alterations in the gene sequence of phages and a wide range of bacterial strains to introduce new strains applied not only for the prevention or treatment of an infection but also for the diagnosis of it [3, 4]. Various mutations, transformation, conjugation, protoplast fusion, electroporation, recombination technology, and molecular genetics are commonly used in genetic engineering. In the past and before molecular genetics methods were invented, researchers used mutations induced by UV radiation and chemicals [5]. Through a combination of biotechnology and genetic engineering science, there are three gene-editing tools including zinc finger nucleases (ZFNs) technology, transcription activator-like effector nucleases (TALEN) technology, and clustered regularly spaced short palindromic repeats-CRISPR associated (CRISPR-Cas) technology as first, second, and third generation technologies in recent years for gene editing by double-strand breaks in desired sequence of genes. The ZFNs and the TALEN are restriction endonuclease-based systems that show limitations such as inducing nonspecific mutations and are time-consuming, expensive, and nonspecific methods [6, 7], while the CRISPR technology is a more efficient and powerful editing method with more flexibility and simplicity [8].

The widespread use of antibiotics in medicine, veterinary, and agriculture causes the emergence and spread of antibiotic-resistant bacteria, so many infections will become untreatable [9, 10]. The World Health Organization (WHO) estimates that 10 million people will die from infections a year by 2050 [11]. One of the applications of GEMs is their potential activity against resistant to antibiotics bacterial pathogens. Two biochemists Herbert Boyer and Stanley Cohen developed the first GEM in 1973. They designed a new plasmid harboring antibiotic resistance gene and then transferred it to *Escherichia coli* (*E. coli*). The result showed that in vitro-produced antibiotic-resistance plasmid is active biologically and functionally in transformed *E. coli* [12]. This work was followed by Yanish and Mintz's research by which this method was applied in animal models [13]. Microbes are also associated with disorders like diabetes and cancers; however, there is ambiguity in the role of probiotics or microbiota in the pathogenesis of disease. Another application for GEM is seen in vaccinology. Vaccines are the main strategy for preventing most infectious diseases. When parts of the genome of pathogenic microorganisms are underchanged, the weakened nonpathogen strain is created. In addition, nonpathogenic bacteria can be changed by genetic engineering to express antigens on their surface, resulting in stimulation of the immune system. Such GEMs provide long-lasting immunity and stable protection in constructed vaccines such as recombinant vaccinia virus and herpesvirus of Turkey (HVT) modified to prevent rabies and Marek's diseases by vaccination. Besides, microorganisms can be subjected to genetic engineering to obtain a high number of useful substances from the microbes or the host such as cytokines, enzymes, and bacterial metabolites [14]. Genetic sensors have also been designed for diagnosis purposes and the identification of specific markers in diseases [14]. Compared with drugs and antibiotics, genetically modified organisms represent fewer side effects and better permeability [15]. Considering the ability of GEMs to overcome scientific problems, we will give a brief review of them including phages and bacteria and their effect on the treatment of cancer, disorders, and microbial infections.

## 2. GEMs Producing Technique and Tools

**Figure 1:**
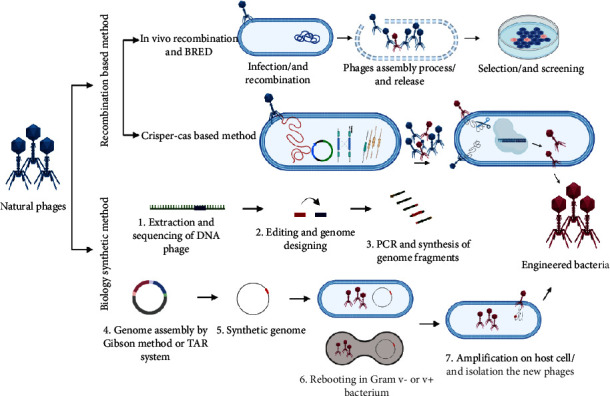
Workflow of phage genome engineering strategies. “Genome assembly and rebooting” is kind of synthetic method and uses phage genome that assembles by smaller and then does the overlapping of DNA fragments through two ways, Gibson method and transformation associated recombination (TAR), using yeast. DNA synthetic transformed into the Gram-negative bacterium (e. coli) or Gram-positive bacterium (L form) to rebooting phage genome. Finally, phages replicate and infect the host and then followed by lysis which, for Gram-negative bacteria, perform by chloroform lysis while it has not effect on Gram-positive bacteria. Therefore, L form bacteria is using which hypotonic pressure select for cell lysis occurred and mutated (or engineered phages) release. Recombination-based methods are divided into two in vivo recombination and BRED and Crisper-Cas based method. Edited templates which were infected in host cell recombine through phage genome replication, and then, mixture of wild-type and recombinant phages was produced. In contrast to synthetic biology method, in last step of BRED and in vivo recombination method, screening is conducted to select certain and appropriate strain by plaque screening method.

There are different methods to modify microorganisms for their application in medicine, agriculture, and industry (Figure 1). In microbial genetic engineering, target genes are first sheared, spliced, and integrated using genetic operation tools before being inserted into chassis cells. Recombinant genes are therefore incorporated into the intended products or provide the bacterium with new phenotypes. The present study discusses novel and widely used methods of genetic engineering of bacteria and phages. Techniques used for the genetic manipulation of bacteria include CRISPR-Cas9, Bacterial artificial chromosomes (BACs), phasmid transfection, phage infection, protoplast fusion, conjugate transfer, and transposition recombination [16]. Small gene fragments (<10 kb) can be modified using Polymerase chain reaction (PCR) or restriction enzyme digestion and direct DNA synthesis [17], while recombination methods such as CRISPR-Cas9 and Red/ET are used for larger sizes (more than 50 kb), which replace, remove, or add gene segments in plasmids or genomes. CRISPR-Cas9 can modify bacteria DNA pieces of up to 100 kb [16]. “CRISPR or clustered regularly interspaced short palindromic repeats” are short and partially palindromic repetitive DNA sequences found in the genomes of bacteria and other microorganisms used to perform targeted genome editing using the adaptive immune system of prokaryotes. CRISPR arrays are inserted into the genome of microorganisms through a process known as adaptation. As a critical component of the immune system, they protect the health of organisms. CRISPR-Cas systems offer microorganisms RNA-directed adaptive protection against invading genetic elements by instructing nucleases to bind and cleave specific nucleic acid sequences [1, 18, 19]. CRISPR-Cas consists of a simple two-component system used for targeted gene editing. The first component of this system is the single-agent Cas9 protein with endonuclease properties, which contains RuvC and HNH domains and is responsible for separating DNA strands. The second component of gene editing is a single guide RNA (sgRNA) carrying a scaffold sequence, which allows it to anchor to Cas9 and place the spacer of 20 complementary pairs of the target gene in the vicinity of the PAM, thus directing the Cas9 protein toward the target gene. Finally, by inserting the CRISPR/Cas9 complex into the cell, double-strand breaks (DSBs) at the target genomic site are created [2]. After recognition and cutting, DNA repair and editing are carried out through cellular mechanisms of the nonhomologous end joining (NHEJ) or homology-directed repair (HDR) pathway. NHEJ is a common error-prone method involving the random insertion-deletion of base pairs (Indel) at the cleavage site. This mechanism typically leads to frameshift mutations, a premature stop codon, and/or the formation of a nonfunctional polypeptide. This method has potential use in genetic deletion experiments, gene disruption therapy, and CRISPR genomic functional screening. HDR is an error-free repair mechanism that uses the homologous section of the unedited DNA strand as a template to repair the damaged DNA, resulting in error-free repair. This method is particularly appealing for clinical applications. In general, this method allows researchers to examine the function of genes to find novel applications in medicine and biotechnology, in addition to precise and efficient modification of bacterial genome [1, 3]. To incorporate foreign DNA segments into the bacterial genome, homologous recombination and transposition are also accessible [1]. When selection markers or CRISPR editing alleles are not available, transposition is the best way to integrate target genes into the bacterial genome [4, 5]. So far, the most often employed transposases are Tol2 [6], Tn7 [7], Tn5 [8], ICEBs1 [9], and sleeping beauty, piggyBac [10], which can integrate very small or large DNA pieces into the genomes of bacteria. However, when the size of the inserted fragments of DNA increases, the transposition efficiency decreases significantly [20].

The transfer methods are determined by the size of the DNA and the properties of the bacterial host. Electroporation and heat shock transfection are common methods for plasmid transfer in *Salmonella*, *E. coli*, *P. aeruginosa*, *B. thuringiensis*, and other bacteria. Plasmids are often transferred from donor bacteria to recipient bacteria via conjugative transfer and protoplast fusion [2]. The conjugative type IV secretion system, for example, works in combination with DNA-processing machinery known as the “relaxosome,” and a huge extracellular tube known as the “pilus” is capable of coordinating directed conjugated plasmid transport [3]. Furthermore, homologous recombination technologies, namely, homologous recombination, site-specific recombination, recombination, and the CRISPR-Cas9 technology, allow for the direct insertion of desired genes into the host genome in a predictable strain. Typical homologous recombination techniques need more than 1 kb homologous sequences to achieve target gene recombination into the chromosomal genome [4]. Nevertheless, the plasmid capacity for carrying and transposition techniques do not permit the operation of big DNA fragments, particularly those greater than 100 kb [5]. So, researchers are more likely to use phage recombination systems, integrase-mediated recombination systems, bacterial artificial chromosomes, and transformation-associated recombination to enable heterologous expression of big gene clusters [1].

Filamentous fungi have attracted the interest of scientists due to their exceptional capabilities as cell factories to produce essential products for humans. There are various broad techniques of genetic transformation for fungi available today, such as *Agrobacterium*-mediated transformation (AMT), protoplast-mediated transformation (PMT), shock-wave-mediated transformation (SWMT), and electroporation, biolistic approach [6]. PMT is a popular fungal transformation approach that involves the use of commercial enzymes to remove complicated cell wall components in order to generate protoplasts. PEG and other chemical reagents enhance the fusing of foreign nucleic acids with protoplasts [7].

*Agrobacterium tumefaciens*, a Gram-negative bacterium, may infect wounded plants and introduce the Ti plasmid, which causes tumors. The Ti plasmid, which is more than 200 kb in size, penetrates the plant through the injury and integrates into the genome of the infected cells. This inserted DNA fragment, often known as transfer DNA or T-DNA, includes genes that code for plant hormones that promote tumor development. The target gene was inserted between the T-DNA boundaries using a binary vector, which transformed the recombinant plasmid into *A. tumefaciens*. The target gene was integrated into the fungal genome using a positive *Agrobacterium* clone [9, 21].

Electroporation provides a simple, quick, and efficient transformation process. Electroporation involves storing electric charges in a capacitor to generate a high voltage, striking the sample with the impulse voltage, and rapidly transferring foreign nucleic acid into cells. In the transformation of fungus, square waves or exponentially decaying waves are typically utilized [6, 10].

Particle bombardment, also known as biolistic transformation, includes foreign DNA adsorption on tungsten or gold particles and is delivered into host cells under extreme pressure. This process can accomplish either steady or temporary changes, with elements such as cell type, growth environment, and density influencing its effectiveness [22, 23]. Particle bombardment is a very effective genetic transformation technology that is not restricted by host or species cell types. It works well for fungi that are problematic to cultivate or make protoplasts from. However, because equipment and consumables are costly, it is only explored when other approaches fail. *Aspergillus nidulans* and *Trichoderma reesei* have been effectively transformed by particle bombardment [6, 13, 14].

SWMT, an energy transmission and transformation technology, causes transitory pressure disturbances and twisting force between cells, resulting in a cavitation effect. It is utilized in medical procedures such as kidney stone crushing and orthopedics. SWMT increases the permeability of cell membranes, allowing exogenous nucleic acid to get into cells. In addition to successfully introducing foreign nucleic acid into *E. coli*, *S. typhimurium*, and *P. aeruginosa*, this approach has also been applied to fungi such as *A. niger*, *Phanerochaete chrysosporium*, and *Fusarium oxysporum* [6, 24, 25].

Many viral components are modified genetically for utilization in biomedicine, nanotechnology, or biotechnology. In the majority of cases, the protein engineering strategies have relied on knowledge-based genetic modification of the viral particle to give heterologous proteins or peptides on the capsid surface (or involves them in the capsid cavity) through integrating the (poly) peptides as a lengthening of a free terminal end of a capsid protein, provide heterologous peptides on the capsid surface by integration in exposed loops, and replace one or a few unnecessary residues in a capsid protein in order to create new sites for heterologous inorganic, organic, or biological components to bind covalently or noncovalently, or less frequently to alter an intrinsic feature or function of the virus particle itself [26].

As shown in Figure 1, there are two general strategies for creating engineered phages. (1) In infected cells, wild-type genomes can recombine using a DNA-editing template. (2) Smaller fragments assemble to form a larger synthetic fragment and then reactivate to generate progeny [12]. A natural biological process known as homologous recombination involves the exchange of nucleotide sequences between two DNA molecules with similar or identical sequences. It was applied in the first generation of phage genome engineering techniques. Parental phages with different phenotypes were coinfected with host cells through the process named phage crosses, and then recombination occurred between the genomes of two phages. Finally, the progeny phages were screened for wanted phenotypes. Recombinants with the right phenotypes were then isolated for further investigation [13, 14]. Phage crosses can only combine existing phage genomes and need markers and phenotypes to determine recombinant phages [24]. To address this limitation, donor plasmids were utilized for insertions, deletions, and replacement of nucleotides in the phage genome. A plasmid with a planned mutation flanked by homologous phage sequences is developed and introduced into a bacterial host and subsequently infected with the phage [13]. Notably, the low frequency of recombination in homologous recombination and the need for processes for screening phages with the desired phenotype are two limitations of this approach [27]. Recombineering is a technique for increasing recombination efficiency by using temperate phage native recombination mechanisms that require the production of phage recombination proteins such as Rac RecE/RecT and lambda Red within the recombination host, which protects the editing template from degrading and facilitates annealing with the injected phage genome [26, 27]. The bacteriophage recombineering of electroporated DNA (BRED) method needs coelectroporation of the donor DNA and phage DNA template into bacterial cells that express RecE/RecT-like proteins through plasmid or chromosomally inserted genes [26, 28, 29]. The donor DNA has the necessary mutations flanked by engineered phage homologous sequences, which causes the phage genome and donor DNA to recombine in a homologous manner. So, recombination only occurs once phage genome replication has started. As a result, both wild-type and mutant phages would be recovered and found in the produced plaques [13, 28]. In conclusion, recombineering-based approaches have the potential to be used in a variety of bacterial species and allow precise mutation of phage genomes at a significantly better rate than standard homologous recombination [30]. Another method based on recombination is the “CRISPR-Cas strategy” which is also harnessed for genome engineering purposes in phages [13, 31]. Cas9 protein, crRNA, and trans-activating crRNA (tracrRNA), the three parts of the CRISPR-Cas system, are often cloned onto a single plasmid. The crRNA and tracrRNA can either be expressed individually or as a single fusion RNA [32–34]. Once these components are transformed into the host cells, they form a CRISPR-Cas9 complex which binds specifically to the target site in the phage genome during phage infection, resulting in the creation of a double-strand DNA break. In bacteria, the lack or low efficiency of NHEJ repairing systems makes the cleavage of the CRISPR-Cas9 complex usually lethal to the phage. This is why it is common for the DNA break to be repaired by recombination with the donor to generate mutants of interest when the homologous donor is provided. Therefore, the CRISPR-Cas system is a powerful tool for the precise editing of genomes, as it can target specific genes or regions and induce the desired mutations [13, 34–36]. The “rebooting method” is another phage engineering strategy. The acquisition of active virion from the phage genome is referred to as phage rebooting. Synthetic approaches for genome assembly outside of natural bacterial hosts have been developed to address the issue that phage gene products may be hazardous to their bacterial hosts. These methods depend on the transformation of small to medium-sized fragments of DNA into complete phage genomes via transformation-associated recombination (TAR) or in vitro enzymatic assembly (Gibson assembly) [27, 37, 38]. The synthesized phage genome can be reactivated by transforming into suitable host cells, L-forms, and cell-free expression systems [30]. In the TAR technique, several large DNA segments recombine in yeast artificial chromosomes (YAC) [33]. Researchers have altered and rebooted several Gram-negative and Gram-positive bacterial phages, including *Klebsiella* phage K11, *E. coli* phages T3 and T7, *P. aeruginosa* phages, and *Listeria monocytogenes* (*L. monocytogenes*) phage P35, based on the assembling and capturing of synthetic genomes into YAC [37, 39–41]. Gram-positive bacteria often have low transformation efficiency. A recent study found that employing L-form bacteria efficiently reboots phages of Gram-positive bacteria. L-form bacteria do not have a cell wall and, unlike their parent cells, can take in enormous amounts of DNA such as phage genome DNA. It was demonstrated that L-form *Listeria* may be used not only to reboot *Listeria* phages but also to reboot *Staphylococcus* and *Bacillus* phages [13, 40].

New synthetic biology approaches for rebooting phage genomes outside of host cells remove the necessity for DNA transformation and allow to produce phages that infect unfamiliar or undescribed hosts. Using cell-free transcription-translation (TXTL) methods, high yields of self-assembling T4, T7, MS2, and FX174 phage particles were synthesized in a test tube using optimized *E. coli* extracts and a modest amount of phage DNA. Although previous genetic engineering was limited to phages infecting extensively studied lab hosts, the recent development of cell-free systems from more bacteria is expected to expand future phage engineering to novel bacterial hosts such as *Streptomyces*, *Vibrio*, *Pseudomonas*, and *Bacillus* species [27, 42–45].

### 2.1. Genetically Engineered Viruses

Viruses, microscopic organisms that live in humans, animals, and plants, can cause devastating infections and reduce crop output and product quality in agriculture, threatening population nutrition, fiber production, and medicines [46]. Viruses, such as smallpox, influenza, and AIDS, have had a major effect on human history and are often seen as enemies. On the other hand, advances in biotechnology and next-generation sequencing technologies have accelerated their discovery, identification, and manipulation, making them important instruments for various biotechnological applications [47]. Viruses have efficient machinery and genetic structures that allow them to be easily manipulated. Early records of its use date back to the 18th century, when the first smallpox vaccine was developed [48]. In the late 1800s, Louis Pasteur created rabies vaccines, which were later followed by polio, measles, influenza, and rubeola vaccines. Vaccines are just one example of how viruses can be used as beneficial agents [48, 49].

Viruses may be genetically altered for a number of purposes, including gene therapy for the treatment of genetic illnesses, oncolytic viruses, vaccine production, and immune cell stimulation [50]. Viruses may also be utilized as vectors by eliminating their pathogenic components while preserving their gene-delivery capabilities, making them very adaptable agents for carrying and delivering genetic material [48]. In gene therapy, viral vectors such as lentivirus, adenovirus, and adeno-associated virus (AAV) are used to deliver functional genes into human cells. AAV is used in Luxturna, a European Union (EU) approved medication, to restore vision in individuals with progressive visual loss. Another use of viruses in gene therapy is cancer treatment. Viruses target cancer cells, making tumors more apparent to the immune system. Viruses have a wide range of biotechnological applications, such as medicine, pharmacology, agriculture, and materials industry. Plant viruses are utilized as vectors for particular protein expression or virus-induced gene silencing, which inhibits homologous gene expression and results in function loss [48, 51, 52]. AAV vectors are the most widely used vectors for gene transfer in the treatment of a wide range of human disorders. AAV vector-mediated gene transfer was recently licensed for the treatment of hereditary blindness and spinal muscle atrophy, and long-term therapeutic outcomes for other uncommon illnesses such as Duchenne muscular dystrophy and hemophilia have been established [53].

Baculovirus expression vectors are utilized in eukaryotic cells to produce complex glycoproteins. Baculovirus biology studies benefit from genome editing of single-copy baculovirus infectious clones (bacmids). Bacmids, on the other hand, are not commonly employed because of the ease with which genes of interest might be lost. Pijlman et al. discovered that relocating the attTn7 transgenic insertion site avoids gene deletion, leading in increased levels of protein expression [54]. The scientists developed a new bacmid to successfully generate chikungunya virus-like particles for commercial vaccinations, indicating substantial advances in the utilization of bacmids as expression vectors. Hsu et al. designed a polycistronic baculovirus expression vector to produce virus-like particles (VLPs) harboring various parts of Porcine epidemic diarrhea virus (PEDV) in pigs in order to elicit immunization against PEDV [48, 55]. Maeda et al. made significant advances to the use of viruses to improve plant breeding. The authors exploited the *Arabidopsis* flowering locus T gene to induce flowering in grapevine (virus-induced flowering, VIF); this work demonstrates the potential of ALSV vectors as VIF to decrease the generation period of grapevine seedlings [56].

As mentioned, another use of genetically engineered viruses is in the treatment of cancer. Recently, oncolytic viral therapy has been identified as a potentially effective new therapeutic strategy for the treatment of cancer. A naturally occurring or genetically modified virus that may selectively reproduce in cancer cells and destroy them without endangering healthy tissues is known as an oncolytic virus [57]. JX-594, also known as pexastimogene devacirepvec or Pexa-Vec, is a genetically modified vaccinia virus that possesses an insertion of the human granulocyte-macrophage colony-stimulating factor (GM-CSF) gene to enhance the antitumor immune response and a mutation in the TK gene that confers cancer cell-selective replication. A marker LacZ gene insertion is also present in JX-594 [58–60]. Ramesh et al. created the oncolytic adenovirus known as CG0070 [16]. The human GM CSF gene is integrated into the Ad5 adenovirus by engineering that drives the human E2F-1 promoter to drive the E1A gene. The retinoblastoma tumor suppressor protein (Rb), which is frequently altered in bladder cancer, regulates E2F-1. A lack of Rb binding causes an E2F-1 that is transcriptionally active. According to studies, engineered oncolytic viruses can induce antitumor immune response in addition to their oncolytic activity [57]. Some oncolytic viruses can be designed to produce therapeutic genes or to functionally modify cancer-associated endothelial cells, allowing T lymphocytes to be recruited into immunological-excluded or immune abandoned tumor microenvironments [17]. Additionally, the measles virus has been genetically modified to generate a single-chain antibody that detects carcinoembryonic antigen (CEA), a tumor antigen that is expressed preferentially on some adenocarcinomas [18].

Also, genetically engineered viruses have been used in the field of vaccine production. Parker et al. employed genetically engineered, conditionally replicating herpes simplex virus (HSV) vaccine candidates that express either interleukin-12 (IL-12) or GM-CSF to protect against HSV infection and/or illness. The result of this study showed that animals previously immunized with these candidate vaccines demonstrated dose-dependent protection after intracranial, intraperitoneal, or intranasal challenge with the highly virulent E377-MB wild-type HSV-1 and Latent virus was not identified at a greater incidence in animals vaccinated and then challenged with E377-MB than in animals immunized alone. These findings imply that cytokine-expressing, conditionally reproducing HSV may induce protective immune responses and remain safe in an experimental mouse model [19]. In general, it can be said that by using genetic engineering, even viruses, which are always thought to be dangerous microorganisms, can be used in industry and medicine.

## 3. Genetically Modified (GM) Phages

Bacteriophages have been effective in the treatment of bacterial infections [61, 62]. Phages are known by unique features including specificity, narrow mode of action, safety and tolerability, easy administration, selectivity, and less expense. These characteristics make phages to be considered as alternative therapy for treatment of bacterial infection [63]. There are limitations described for phage therapy such as specific target, narrow spectrum action of phages than antibiotics [64], and issues related to the formulation and stability of phages as a pharmaceutical agent. There is not enough information about the biology of phages, highlighting a need for extensive studies in this regard [65, 66]. One of the strategies to overcome limitations is to create novel GM phages with therapeutic capabilities by engineering technology like GMPs [64] mediating modification and restoration of the gut microbiome. The human gut microbiome comprised of bacteria, viruses, and archaea plays an important role in both human health and disease states [63, 67]. Dysbiosis and imbalance in the microbial composition of the gut microbiota are related to diseases like irritable bowel syndrome (IBS), inflammatory bowel disease (IBD), coeliac disease, obesity, cardiovascular disease, and asthma [68]. Using phages to restore this imbalance is considered a promising therapeutic method. Hu et al. treated a germ-free mouse with lytic phages to colonization with commensal gut bacteria. They found the phages destroyed the sensitive strains in the gut, while 68% *Enterococcus faecalis (E. faecalis*) became resistant to the phages in follow-up [69]. In spite of the major therapeutic potential of natural phages, bacteria can become resistant to phages and the immune system can trigger against phages. Companies tend to develop GM phages through specific techniques like the CRISPR-Cas system for therapeutic purposes targeting pathogenic bacterial strains and eukaryotic cells(Table 1), increasing the susceptibility of resistant bacteria to antibiotics, increasing the host range of phages, and establishing homeostasis in the microbiota [63, 85]. CRISPR-Cas is used for the development of GM phages by targeting and removing undesirable genes like antibiotic-resistant genes. The engineered M13 phage targets bacteria carrying the beta-lactam-resistant gene, leading to a reduction in the number of living bacteria cells. Similarly, a reduction in the number of living bacterial cells was observed when fluoroquinolones-resistant *E. coli* strains harboring a *gyr* A mutation were treated with CRISPR-GM phage [86]. The limited host spectrum of phages results in advantages and disadvantages. Since treatment with a single phage would not be effective in multimicrobial diseases [87], preparing a phage cocktail and phage engineering are considered strategies to increase the host range of bacteriophages [88]. The host spectrum of the phage can be expanded using targeted mutagenesis in the tail fiber regions involved in determining the phage host [63, 89]. One of the reasons for persistent infections is bacterial biofilm formation on healthcare devices. The extracellular matrix of biofilms acts as a shield to prevent antibiotic penetration into the biofilm. Some of the GM phages carry enzymes to destroy this matrix and consequently facilitate the penetration of antibiotics into the biofilm [90]. Besides, GM phages are used as drug carriers to treat Alzheimer's and Parkinson's diseases and cancer [63]. Bar et al. investigated the impacts of hygromycin-conjugated GM phages on human breast adenocarcinoma in SKBR3 cell line. They reported that hygromycin had a better effect by 1000-fold, compared with conventional drugs [91]. The blood-brain barrier (BBB) acts as a hurdle against access to therapeutic peptides, antibiotics, and chemotherapy agents. Using the “Trojan horse” strategy, phages have successfully carried drugs across the BBB. In a study conducted by Anand et al., *Salmonella typhimurium* (*S. typhimurium*) bacteriophage P22 was genetically engineered to express a peptide on its capsid that enables the phage to pass through the BBB. Results of this showed that the Ziconotide peptide is well expressed in snail venom and has analgesic properties [92]. Shiga-like toxin produced by EHEC causes inflammation in the intestine, resulting in an increase in proinflammatory cytokines such as IL-6 as well as IgG1, IgG2a, and IgA levels due to the activation of the immune system. Therefore, following the use of the engineered *λ* phase, IL-6, IgG1, IgG2a, and IgA levels in the serum are significantly reduced [93]. Recently, a case report of *Mycobacterium abscessus* (*M. abscessus*) was analyzed in a 15-year-old cystic fibrosis patient with an engineered cocktail of three phages. Lytic derivatives effectively killing *M. abscessus* were developed in ZoeJ phage through genetic engineering by the BRED method. Intravenous phage therapy was well tolerated and linked to clinical symptom improvements such as sternal wound healing, better liver function, and significant clearance of infected skin nodules [94]. Moreover, engineered phages have the ability to degrade biofilm exopolysaccharide. Through genetic engineering, T7 and Y2 phages express two genes, dispersin B (*dsp*B) and amylovoran depolymerase (*dpoL*1), encoding exopolysaccharide-degrading enzymes, thereby increasing cell lysis inside the biofilm [64]. *Clostridium difficile* (*C. difficile*) as one of the important nosocomial pathogens causes high mortality and morbidity rates. The current treatment for *C. difficile* infection is the use of broad-spectrum antibiotics; although the treatment is successful, recurrence occurs in 30% of cases. Since no lytic phage is known for *C. difficile*, the lysogenic phage is considered to target *Clostridium* by using the bacteria's own CRISPR system for degradation of the bacterial genome. *C. difficile* CD24-2 phage has been engineered to deliver the type I-BCRISPR-Cas system of *C. difficile*. The phage can target *C. difficile* by using the bacteria's own CRISPR system to degrade the bacterial genome. Bacterial death occurs simultaneously through bacterial genome degradation by CRISPR-Cas system and the expression of lytic genes of bacteriophage, holin, and endolysin [95].

### 3.1. Genetically Modified (GM) Fungi

Because fungi are a good source of secondary metabolite like isoprenoids, nonribosomal peptides, alkaloids, and polyketides as natural products, it found good place among microorganisms. In the past, fungi were the sources of production of antimicrobial and anticancer agents for diseases such as tuberculosis and gastritis and treatment of kidney disorders. Today, it was found that these properties are due to the presence of secondary metabolites [96]. These compounds which have medicinal properties can undergo modification to be substituted with the desired secondary metabolite by using various laboratory techniques such as the recombination method [97].

With the development of molecular biocellular science and biotechnology and the introduction of genetic engineering tools, it is possible to strengthen the efficiency and usefulness of fungi, e.g., by increasing their capacity to produce useful substances [98]. In short, techniques such as homologous and heterologous expressions [97], protoplast fusion technology, electroporation, shock-wave, and biolistic approach are involved in genetic transformation for fungi [6]. Among them, transformation is the first introduced and main method of genetic alteration in fungi [98] in which protoplast-mediated transformation (PMT) or protoplast fusion technology is one of the most important methods of genetic material transport in fungi by transformation [99]. In this method, following the removal of the cell wall with the help of enzymes, the membrane-covered protoplast is released. In the meantime, if calcium ion is used, the penetration of DNA from the membrane into the mushroom is facilitated.

Unlike bacteria, transformation has limitations due to the firmness of the cell wall. Unlike bacteria, transformation in fungi is limited by the firmness of the cell wall. Furthermore, certain fungi lack cell walls altogether, presenting challenges to traditional transformation methods. To overcome these limitations, alternative transformation techniques such as agrobacterium-mediated transformation (AMT), polyethylene glycol (PEG)-mediated transformation (PMT), electroporation (EP), biolistic transformation, and lithium acetate mediated transformation are utilized. [97]. In these methods, the mediating agent such as *Agrobacterium tumefaciens* is used in AMT as a carrier to transfer the genetic material from the donor to the host [100]. Genetically engineered fungi have been investigated in many studies, such as entomopathogenic fungi, which were produced as a nature-friendly strain with the aim of replacing chemical insecticides, and will be very important in the agricultural field [101]. Among the fungi, filamentous fungi were widely welcomed because they are known as cell factories for the production and secretion of proteins. The revelation of the metabolic pathway and physiology of filamentous fungi accelerated the genetic modification of these organisms [6]. For explaining GM fungi, filamentous fungi are discussed for reasons such as, first, almost half of the commercially available proteins were synthesized by filamentous fungi, second, many filamentous fungi classified as generally regarded as safe (GRAS) strains are known, and third, it also has a great capacity to produce and secrete proteins that can be used in industry and medicine [102].

Choosing a host with high transformation power and a suitable marker is one of the important items of genetic engineering, which not only reduces the risk of creating false transformations but also increases the final product [103]. Wild strains are mostly used in genetic engineering due to understating of their genome sequence being known [99]. Modified strains such as strains with defects in protease that are used in protein expression can also be good targets [104]. Auxotrophic marker selection can be a criterion in choosing a suitable host for genetic engineering such as the hygromycin gene and the gene encoding orotidine-50 monophosphate decarboxylase pyrG, respectively, as the most important and common markers of antibiotic resistance gene and auxotrophic gene in filamentous fungi such as *Aspergillus fumigatus* (*A*. *fumigatus*) and *Aspergillus terreus* (*A. terreus*) [105]. However, markers related to antibiotic resistance are not always responsive because some strains can be resistant and cannot be used in transformation [106]. As much as these markers are limited, auxotrophic markers which are known as essential metabolic genes of protein biosynthesis are mostly used in genetic engineering and production of transformed strains [107].

## 4. GM Probiotics

**Figure 2:**
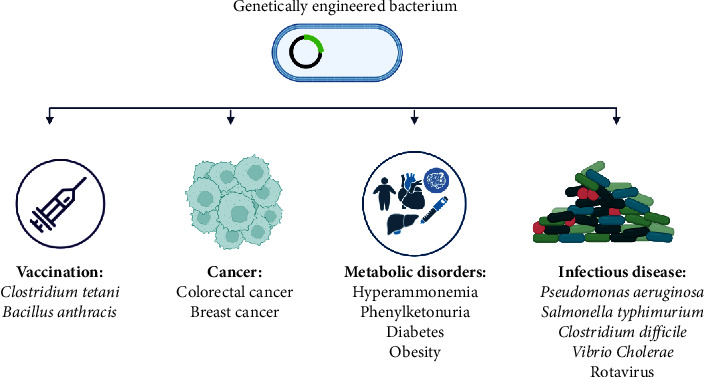
Medical applications for genetically engineered bacteria (GEB). Infectious disease: GEB can combat bacterial infections by (1) the release of toxins and toxins neutralization and (2) the production of QS components that lead to the expression of surface adhesion preventing pathogenic colonization and production of antimicrobial factors mediating bacterial killing. Also, GEB can be designed to secret antibody-like fragments to prevent pathogenic bacterial adhesion to host cells. Metabolic disorders: GEB can release antibody fragments against pro-inflammatory cytokines, anti-inflammatory cytokines, antioxidants, or certain enzymes. Cancer: GEB (1) can accumulate and replicate in cancerous cells and subsequently express certain bacterial toxins, the converting enzymes, pro-cytokines, and apoptosis inducer molecules. (2) GEB can harbor plasmid encoding shRNA for silencing genes after transformation into cancerous cells. Vaccination: GEB (1) can promote immune cell recognition and uptake of antigens through the expression of intracellular/ surface antigens via bacteria chassis as an adjuvant, (2) designing and engineering an antigen by dendritic cells-targeting peptides, and (3) packaging antigen into the outer membrane vesicles for enhancing immune cell recognition and uptake of recombinant antigen.

Probiotics are live microorganisms that excrete beneficial effects if administered in insufficient quantities [108]. They have attracted the attention of researchers in the treatment of diseases since antibiotic resistance among bacteria and no longer effectiveness of antibiotics became a concern [109]. Probiotics are useful in the prevention and treatment of human and animal diseases. Considering that the impacts of probiotics are significantly related to the type of species and its prescribed dose, their performance can be attributed to their ability to inhibit the colonization of pathogens, establish homeostasis in the microbial flora, and modulate the immune response and metabolic pathways [110]. Some probiotics such as lactic acid producer strains involved in endocarditis and bacteremia in vulnerable patients or the enterococcal probiotic strains are too risky due to the presence of main virulence genes and antibiotic resistance issues [111, 112]. The biggest limitation of probiotics is their possible weakness in survival through passaging and reaching target tissues by the acidic environment of gastrointestinal tract, oxygen levels, and their survival in food packaging. The size and number of probiotics required for effectiveness, source of isolation of probiotics, and their reaction with the normal microbial flora are also concerning issues [113, 114]. Genetic engineering can be helpful in the reduction of probiotic pathogenicity and reinforcement of their useful properties. In addition, genetic engineering can make probiotics safe for human applications such as vaccination and delivery of target proteins and drugs (Figure 2) [110]. Bioengineered probiotics would be more useful in the diagnosis and treatment of specific diseases (Table 2) [3]. There are several engineered probiotics in different stages of clinical trials (Table 3).

### 4.1. Criteria for Selection and Safety Issues of GM Probiotics

Four important criteria for the selection of probiotics are necessary to consider for the development of GM probiotics, including safety, technological, functional, and physiological fitness. In relation to the safety of probiotics, selected probiotics should not only have a good history of safety but also be isolated from the gastrointestinal system of healthy people. From the technological view of preparing probiotics, the ability to grow massively during bacterial cultivation, to survive probiotics during preparation, and to storage is very important. Besides, a selected probiotic strain should be stable and exhibit genetic stability. In terms of functionality, probiotics should be able to adhere to cells, be in balance with the normal human flora, and well grow in the target organs. Finally, probiotics physiologically should be able to metabolize cholesterol and carbohydrates, modulate the immune response, and prevent the growth of pathogens antagonistically [159, 160]. Since GM probiotics have been subjected to genetic modification such as gene addition, changes in immunogenicity, and metabolic pathways, their safety and persistency in the surrounding environment should be examined by safety tests [110, 161].

### 4.2. Clinical Application of GM Probiotics

GM probiotics by expressing heat-shock proteins such as GroES and GroEL are able to tolerate stress in a wide range of temperatures [162], by delivery of therapeutic antimicrobial peptides (AMPs) are effective against antibiotic-resistant bacteria [163], and by targeting tumor cells and replication in the tumor site are useful to treat cancers exhibiting resistance to traditional cancer therapy [164]. Moreover, understanding the gut-brain axis, the connection between the GI and the brain by the vagus nerve has helped to determine the association of microbial flora and stress, behavior, and mental health. GM probiotics by reducing neurotoxic compounds such as indole and the production of serotonin are effective in cognitive health [165]. Finally, GM probiotics were also used in vaccines by delivering immunogen compounds, which overcome vaccination-associated problems using a weakened pathogen [166].

Engineered-modified *Lactobacilli* spp. may be involved in reducing the symptoms of hyperglycemia for diabetes. In the diabetic animal model designed by Duan et al., GLP-1 (glucagon-like peptide-1) was expressed in *Lactobacillus gasseri* (*L. gasseri*). In diabetic rats receiving orally engineered *L. gasseri,*insulin-producing cells were produced in sufficient quantity and the normal function of insulin-producing cells was no longer disturbed [167]. Other *Lactobacilli* spp. were also subjected to genetic modifications including *Lactobacillus lactis* (*L. lactis*) and *Lactobacillus casei* (*L. casei*). In vitro studies of *L. lactis* were designed to deliver therapeutic proteins like antienterococcal peptides, hiracin JM79, enterocin A and enterocin P, SCI-59, and flagellin, which showed to be effective in the treatment of *E. faecalis* infection, diabetes, and enteropathogen infection, respectively [168–170]. *Enterococci* were not able to grow and survive in the presence of engineered *L. lactis* producing antienterococcal peptides [168]. IBD is influenced by engineered *L. lactis* and *L. casei* genetically modified to deliver Elafin. Elafin is a type of protease inhibitor involved in protecting gastrointestinal surfaces against any damage. According to the result of the examination of the cell line and mouse model, both probiotics colonize in the intestine and successfully produce Elafin [171]. Rosberg et al. designed a recombinant *Lactobacillus paracasei* (*L. paracasei*) to produce linoleic acid isomerase which has a role in fatty acid accumulation. Histological examination of liver tissue in mice model showed higher levels of cis-12 and trans-12 which are directly associated with successful expression of linoleic acid isomerase encoding gene in engineered *L. paracasei* [172]. In the same study by Koo et al., *L. paracasei* underwent genetic manipulation against *L. monocytogenes* infection. Recombinant *L. paracasei* which expressed *Listeria*'s adhesion protein showed significant decreases in invasion and attachment of *L. monocytogenes* in cell line experiment [173]. In another report, *Lactobacillus jensenii* (*L. jensenii*) showed an in vitro anti-human immunodeficiency virus (HIV) effect following genetic modification to secret CV-N, a HIV-1 entry inhibitor cyanovirin-N. *L. jensenii* expressing antiviral peptide reduces the infection in further examination on animal model [174], and there are no inflammation and potential adverse effects following colonization of recombinant *L. jensenii*. Also, modified *L. casei* expressing *Listeria*'s adhesins was able to colonize the intestine and compete with *Listeria* to reduce infection caused by *Listeria*. They mediate the immune system by increasing regulatory and natural killer cells [175]. *Bifidobacterium* spp. are Gram-positive bacillus of obligate anaerobes resident in the gastrointestinal system such as the intestines of mammals. So far, ten types of *Bifidobacterium* spp. have been introduced in humans. These bacteria well colonize the tumor region and survive, suggesting *Bifidobacterium* spp. as a promising candidate in cancer therapy [176]. Several studies highlighted the gene delivery by bifidobacteria in the treatment of cancer. Wang et al. designed an engineered *Bifidobacterium breve* (*B. breve*) strain by electrotransformation of IL-24 gene which was expressed on the probiotic surface. Inhibitory effects on tumor progression were observed by analyzing tumor growth and apoptosis induction, which indicates recombinant *B. breve*-IL24 is a promising strategy in cancer therapy [177]. Wei et al. performed an experiment on the mouse model with colitis by transfer of *Bifidobacterium longum* (*B. longum*) delivering rhMnSOD (recombinant human manganese superoxide dismutase) to colitis mouse model [178]. The reduced effects on the symptoms of colitis as well as histological findings showed that *B. longum* can be a good delivery candidate for the treatment of colitis. Genetically engineered *E. coli* Nissle (EcN) has shown its beneficial effects in both infections and diseases. Duan et al. used engineered *E. coli* Nissle expressing CAI-1 in a mouse model suffering from *Vibrio cholerae* (*V. cholerae*) infection. They found that the binding rate of toxin to the intestine of mice and the number of *V. cholerae* decreased by 80% and 69%, respectively [134]. In the same study by Hwang et al., modified *E. coli* Nissle was used producing S5 pyocin, E7 lysis protein, and DspB to protect the mice model against *Pseudomonas aeruginosa* (*P. aeruginosa*) infection [85]. They successfully reported that engineered *E. coli* Nissle is a suitable probiotic candidate for treatment and prophylaxis against bacterial infection induced by photogenic *Pseudomonas*. Genetically engineered *E. coli* Nissle harboring HIV-gp41-hemolysin was examined for HIV infection [145]. Colon histological examination and the immunocytochemistry (ICC) analysis indicate successful colonization of colorectum by modified *E. coli* Nissle in a murine model for months while expressing antiviral peptides. This result makes *E. coli* Nissle the first promising live antiviral probiotic against HIV infection. In addition to microbial infection, disorders can also be alleviated by genetically engineered probiotics. Engineered *E. coli* Nissle was investigated for delivery of fructose dehydrogenase and mannitol-2-dehydrogenase enzymes to examine hepatic steatosis disorder in rats [179]. Following administration of engineered *E. coli* Nissle, lipid peroxidation reduced significantly while the serum and hepatic antioxidant enzyme levels were increased. Therefore, engineered *E. coli* Nissle also confer a good probiotic strain in the treatment of metabolic disorders. *Saccharomyces boulardii* (*S. boulardii*) undergoing genetic modification by Chen et al. to prevent *Clostridium difficile* (*C. difficile*) infection (CDI) can successfully neutralize *C. difficile* toxins by secreting a protein called ABAB. This probiotic showed beneficial and preventive effects on animal death in mouse models [180].

## 5. Genetically Modified Other Bacteria

Probiotics and bacteriophages with a long history of safe use for consumers as therapeutic agents and their role in the prevention and treatment of many diseases have already been mentioned. In addition, other engineered bacterial strains have been designed to respond to environmental signals, especially bacterial strains previously displaying no susceptibility to genetic changes have increased. For example, design of *Clostridium* spp. with the ability to produce an anti-inflammatory metabolite, *β*-hydroxybutyrate, overcomes challenges of oral delivery including survival in exposed to stomach acids, enzymes, and bile salts [137, 181–184]. Design of engineered *E. coli* with the potential to treat solid tumors in preclinical models has been reported in several studies. Chowdhury et al. engineered an *E. coli* strain to release an anti-CD47 antagonist nanobody inducing tumor regression and abscopal effects and exhibiting long-term survival in a syngeneic tumor mouse model [185–188]. In addition to *E. coli* bacteria, which have a long history in cancer treatment, anaerobic microorganisms such as *Bifidobacterium* strains are used in preclinical cancer treatment through converting the nontoxic compound 5-fluorocytosine into the cytotoxic compound 5-fluorouracil [189]. Administration of CD-expressing *Bifidobacterium infantis* with the nontoxic compound 5-fluorocytosine significantly inhibited tumor growth in mice [190]. In a study by Yujie Sun and colleagues, it was shown that *S. typhimurium* engineered using *Vibrio vulnificus* (*V. vulnificus*) flagellin B (*Fla*B), which is a natural ligand of Toll-like receptor 5 (TLR5), strongly inhibits tumor growth and is an excellent aid for cancer immunotherapy [191].

## 6. GEMs for Diagnosis and Delivery Purposes

There are different therapeutic molecules such as antibodies, proteins, and biochemical compounds delivered by genetically engineered microorganisms which will be discussed in therapeutic applications section of each genetically engineered organism. In this part, only diagnosis and production of various proteins are discussed [192]. One of the strategies for changing organisms is the use of synthetic biology and various genetic platforms by using which we can genetically engineer organisms [193]. Microorganisms can be genetically modified to be considered as biosensors which can identify specific markers such as chemical substances and molecules, gases, and ions present in various diseases. For instance, *E. coli* has been genetically engineered to identify biomarkers such as glucose and nitric oxide in inflammatory conditions and diabetes [151]. Engineered *E. coli* Nissle, which belongs to probiotic strains, plays major role in the diagnosis of gut inflammation, colitis, and gastrointestinal bleeding through identifying Nitrate [194], Thiosulfate [136], and Heme [195], respectively. In addition, metastasis in liver cancer was diagnosed by *E. coli* Nissle in mice [196]. Other probiotic bacteria such as *Lactococcus lactis* (*L. lactis*) and *Lactobacillus reuteri* (*L. reuteri*) can serve similar function for diagnosis of cholera and *Staphylococcus aureus* (*S. aureus*) infection via sensing CAI-1 (cholera autoinducer-1) [115] and AIP-I (autoinducer peptide I) [116], respectively. Wu et al. developed a new whole-cell biosensor that responds to Quorum sensing (QS) signal molecules to detect bacterial infections (*P. aeruginosa* and *Burkholderia pseudomallei* (*B*. *pseudomallei*)). The results indicated that designed whole-cell biosensors can detect waterborne infections rapidly and cheaply [135]. Another study used *L. lactis* to detect *E. faecalis*. *L. lactis* can generate and secrete peptides that prevent enterococcal growth and reduce its vitality in the surrounding area of this probiotic. The effectiveness of this modified system against multidrug-resistant *Enterococcus faecium* (*E. faecium*) strains was demonstrated [168]. Lubkowicz and colleagues created *L. reuteri* that detects AIP-I, a QS protein generated by *Staphylococcus* spp., during pathogenesis. Their results showed that the engineered biological sensor could detect AIP-I levels in *S. aureus* under various harsh conditions, and these created sensors for staphylococcal contamination detection in hospitals and drug screening will be helpful [197].

Genetically engineered microbes can be designed to transfer host proteins such as enzymes, cytokines, and important bioactive molecules effective in treatment [198], while the other transfer methods were not safe because most of these molecules were degraded. Considering that some microbes have the ability to pass through the rough condition of the body without decomposing, genetically engineered microbes are resistant to barriers, e.g., the gastrointestinal and digestive enzymes, and do not stimulate the host immune system. Therefore, these microorganisms protect the molecules under harmful conditions of the body [199]. Keratinocyte growth factor-2 (KGF-2), trefoil factor (TFF), and interleukin-10 (IL-10) can play an important role in treatment of disease and can be delivered by genetically engineered microorganisms including *Bacteroides ovatus* (*B. ovatus*) and *L. lactis*, respectively [192]. By phase I research on Crohn's patients in humans [200] and colitis in animal model [201], researchers found that *L. lactis* can be a good carrier for delivering IL-10 [200]. Also, *L. lactis* was successful in transferring IL-17A in cancer mouse model [202], Heme oxygenase-1 in mice with colitis [203], and hTFF1 in hamster with oral mucositis [204]. *Lactococcus casei* (*La. casei*) was designed to express human lactoferrin (hLF) as an antibacterial agent which showed significant clearance of *E. coli* by enhancement in phagocytosis and iron depletion in murine model with bacterial infection [205].

Engineered microbes can also be considered as carriers for vaccine components. The recombinant bacteria secrete specific antigens or antibodies to host cells. Sometimes, transport of autoantigens may be associated with tolerance pheromone, resulting in prevention of autoimmune disease [206]. Probiotic strains especially *L. lactis* have been often investigated in studies on infectious and metabolic diseases. Recombinant *L. lactis* strains designed to deliver and secret low-calcium response V (LcrV) antigen were observed to reduce in their bacterial number and survival in vaccinated mice against *Yersinia pseudotuberculosis* (*Y. pseudotuberculosis*) infection [207]. Also, orally administered recombinant *L. lactis* expressing hemagglutinin can confer protection against influenza virus challenge in mice [208]. Moreover, genetically engineered *L. lactis* strains harboring and delivering anti-TNF nanobody, ovalbumin, DQ8 gliadin epitope, and GAD65 and IL-10 were used as vaccine against colitis [209], autoimmune diseases [206], celiac disease (30), and type 1 diabetes, respectively [210].

## 7. Genetic Engineering of Antibiotic Producers

With the increase in bacterial infections caused by pathogens that are resistant to one or more antibiotics, the world has entered another challenge; according to the statement of the WHO, these challenging pathogens include *E. coli*, *S. aureus*, *P. aeruginosa*, *Acinetobacter baumannii* (*A. baumannii*), *Klebsiella pneumoniae (K. pneumonia*), and *E. faecium* [211]. In addition to being resistant to antibiotics, these mentioned bacteria are able to spread this resistance to other antibiotic-sensitive strains. This is a worrying issue and should be replaced by newer treatment methods. One of these new methods is genetic engineering and synthetic biological sciences, which with the conceptualization and protein engineering and in silico design will ultimately lead to the development of new treatment methods that combat antibiotic resistance [140]. So far, more than 30,000 synthetic antibiotics and 7,000 natural antibiotics have been introduced. Mutation and genetic engineering technology with recombinant DNA tools are widely used to genetically manipulate antibiotic-producing microorganisms to produce more antimicrobial compounds [28]. The main goal of genetic engineering in antibiotic-producing microorganisms is the synthesis of new strains of microorganisms that produce the desired antibiotic in larger quantities by changing antibiotic biosynthesize pathways to improve the quantity rate of antibiotic production and synthesis of hybrid and new modified antibiotics [28]. In this part, how to use synthetic biology to achieve this goal is discussed. In this regard, there are two general paths: using genetic engineering to (1) enhance antibiotic production and (2) modify existing antibiotics, which will be discussed further.

### 7.1. Genetic Engineering and Enhancing Antibiotics Production

If access to the encoding genes of metabolites produced by antibiotic-producing bacteria was possible using genetic tools such as mutation, we could increase the production of the desired antibiotics [160]. For example, genetic modifications were applied to the gene encoding amphotericin by deleting the amphDIII and amphL genes in Marinactinospora thermotolerans (M. thermotolerans). Additionally, the cellular function of the genes involved in the production of the nucleoside antibiotic A201A in Streptomyces nodosus (S. nodosus) were studied. These modifications resulted in improved production of both antimicrobial substances. [30, 212]. Genetic alteration leads to an increase in the biosynthesis of antimicrobial substances by improving precursors and flux of metabolites. Likewise, studies showed a 60-fold increase in precursors from carbapenem antibiotic synthesis pathway in *E. coli* producing carbapenems [31] or an increase in the glycolysis pathway that produces antimicrobial compounds in *Streptomyces lividans* (*S. lividans*) following modifying in the metabolic pathway of carbon flux [213]. Also, the transfer of desired genes to the host or their transfer from one host to another is an example of the achievements of synthetic biology in biofactors, where the host benefits from the production of products expressed from the incorporated genes. This is well shown in a study in which the *cyp* gene was transferred from *Ganoderma lucidum* to *Saccharomyces cerevisiae*. There was an 8% increase in the production of a derivative of 3,28-dihydroxy-lanosta-8,24-dien-26-oic acid as a novel ganoderic acid with antimicrobial activity [33]. Also, the transfer of the bacitracin-encoding gene from *Bacillus subtilis* (*B. subtilis*) to *Bacillus licheniformis* (*B. licheniformis*) was shown in Eppelman et al.'s study [34]. Through the gene replacement of the *srfA* gene cluster encoding the surfactin synthetases by integrating the bacitracin biosynthetic gene using a homologous recombination approach, Eppelman et al. showed increased expression of bacitracin biosynthetase in engineered *B. subtilis* and their self-resistance to bacitracin more than *B. licheniformis*.

### 7.2. Genetic Engineering and Modifying Existing Antibiotics

Microorganisms are intrinsically capable of producing antimicrobial compounds, which are also known as secondary metabolites. The great challenge for these antibiotic-producing species is the low production of these metabolites. Synthetic biology has provided more production of the same antibiotics with more diversity by antibiotic synthesis mechanism. Multimodal enzymatic complexes in biosynthetic cluster genes (BCG) and metabolic engineering confer strategies for overexpressing antibiotics [35].

The multimodal enzymatic complex consists of several enzymes including polyketide synthases (PKS), nonribosomal peptide synthetases (NRPS), and a combination of both NRPS and PKS, which these enzymes give the chemical characteristic to the peptide. Synthetic biology with engineering modifications on these enzymes solves the problem of low production of antibiotics [36]. Considering the wide distribution of vancomycin-resistant *Enterococcus* (VRE), attention has been focused on the biosynthetic pathway of glycopeptide antibiotics (GPAs) to deal with resistance using synthetic biology. Yim et al. conducted scaffolded assemblies of multienzymatic complexes from seven glycopeptide antibiotic (GPA) gene clusters of BCGs. These assemblies were then transferred to Streptomyces coelicolor (S. coelicolor) to enhance the diversity of glycopeptide products. Following this addition to *S. coelicolor*, nine new compounds were reported from which eight showed antimicrobial properties against *E. faecalis* [214]. Another glycopeptide naturally produced by *Streptomyces* spp. in a small amount is called corbomicyn. Under one of the synthetic biology platforms called the glycopeptide antibiotic heterologous expression system (GPAHex), an increase in the expression of genes encoding corbomicyn was reported 19 times compared with its normal level [38]. Ji et al. utilized a combination of NRPS and PKS, by another platform of synthetic biology, top-down method, to increase daptomycin level by more than 40% [33]. Daptomycin produced by *Streptomyces roseosporus* (*S. roseosporus*) at inadequate levels is clinically used for combat against methicillin-resistant *S. aureus* (MRSA) [39]. Metabolic engineering is also used to increase the production and diversity of antibiotics. This method reprograms cellular metabolism to enhance the production of metabolites and metabolically engineers enzymes and metabolite flux that is important in the passage of a metabolite [40]. Sometimes, finding the relationship between the biosynthesis of antibiotics and the intermediate compound helps to enhance antibiotic production. For example, the production of bacitracin from *B. licheniformis* was enhanced in a study. This study showed an increase in the production of bacitracin by using synthetic biology tools such as recombinant base techniques. In this work, the production of bacitracin was investigated in relation to a secondary metabolite called S-adenosyl methionine (SAM) [41].

### 7.3. GM Phages against Antibiotic-Resistant Pathogens

Although phages without genetic changes are capable of fighting antibiotic-resistant microbial infections [42], using genetic engineering to modify phage can develop phages with specific abilities, for instance, usually, the phage is designed to target a wider range of bacterial strains. The advantage of this strategy is to use fewer phages in the cocktails, reduce the preparation and purification proceeding of a large amount of phage, and reduce the rate of development of bacterial resistance following synthetic biology [35]. By high-throughput screening, Yeh et al. found host-range-determining regions (HRDRs) in the T3 phage tail. Using site-directed mutagenesis, they genetically modified the HRDRs to produce synthetic phagebodies. The results from phagebodies showed that they turn out to have a wide range of bacterial hosts that can inhibit bacterial growth for a longer period of time [207].

## 8. The Design, Build, Test, and Learn Cycle of Metabolic Engineering

**Figure 3:**
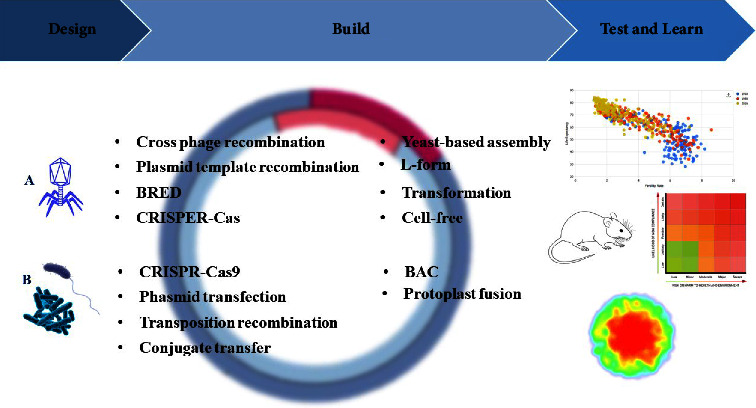
Novel biological systems which composed of four main steps; the Design, build, test and learn (or application) (DBTL) cycle. Following the selection of a certain organism, and determination of the purpose of biosynthesis of GEM, the specific technique was applied to produce a modified organism. The modified organism is being investigated in different clinical trial phases. There are different laboratory and genetic techniques for producing engineered phages (A) and engineered bacteria (B). BRED: bacteriophage recombineering of electroporated DNA, CRISPR-Cas: clustered regularly spaced short palindromic repeats CRISPR associated, BAC: bacterial artificial chromosome.

The design, build, test, and learn cycle (DBTL) as one of the engineering principles is a loop consisting of different stages (Figure 3) and each of them follows a goal to design and introduce a new biological system [43]. Researchers use DBTL to overcome antibiotic resistance problems by producing new antimicrobial agents [35]. In the first stage, Design, problems and challenges were raised and the pathways were determined using high-throughput screening (HTS). In the Build step, all required components related to the host are built. The Test step is for examining all genetically modified constructs toward the target for which they were designed, for example, if it was for partial targeting or the production of a specific protein. Finally, by the Learn stage, researchers learn from the results of the previous step to make a hypothesis [215].

## 9. GEMs for Disease

### 9.1. Engineered Microorganisms for the Treatment of Cancer

The current manner for cancer treatment includes surgery, chemotherapy, and radiation therapy. However, the limitations like the lack of effect on whole region of tumor with anaerobic conditions have led to developing novel treatments based on the transfer of cancer and anticancer antigens or drugs through vectors. As the science of genetic engineering advances, researchers have turned their attention to target tumors using genetically engineered bacteria. There are several types of anaerobic bacteria such as *Salmonella typhimurium* (*S. typhimurium*), *Clostridium*, *E. coli*, and *Bifidobacterium* known as live biotherapeutic products (LBPs) that are able to survive in the tumor site due to their anaerobic nature and adaption to hypoxic condition. They would have many benefits for cancer treatment by their ability to spread and carry the drug, anticancer compounds, proteins, and pre-enzymes to the anaerobic space of the cancer mass [122]. They have many potential benefits for cancer treatment related to their ability to spread and to transfer and release drug or anticancer compounds to anaerobic space of the cancer mass [122, 216]. Following genetic modification of the bacteria to harbor genes and proteins, colonization in tumor site occurred, resulting in not only transfer of antigens or cancer-fighting compounds but also stimulation of the immune response [109, 110, 217, 218]. Moreover, *E. coli* Nissle 1917 can be designed as a targeted transporter to deliver cancer antigens or effective proteins such as p53 and nano-antibodies to the anaerobic areas of the tumor [113]. Selection of an antitumor substance mostly depends on the biogenesis of tumor. For instance, given that amino acid L-arginine is considered as an effective factor in the development of the immune response against tumors, genetic engineering of strains to produce more L-arginine would have a significant effect in preventing cancer. Bacteria-based cancer therapy is the matter that has risen for years [114]. Moreover, probiotics have been considered for their anticancer effects excreting through inducing apoptosis and preventing oxidative stress [159, 160]. As shown in Table 1, it became possible to design new probiotics to fight cancer by genetic technology [109] from which only two genetically engineered probiotics entered clinical phases for treatment of cancers. First, *E. coli* Nissle 1917 was engineered to produce cyclic di-AMP which activates the immune response and the antigen presenting cells (APCs) by affecting on the sequence of interferon [161]. This engineered strain known as SYNB1891 (NCT04167137) currently is under investigation in phase 1 clinical trial. Another antitumor candidate probiotic known as bacTRL-IL-12 (NCT04025307) had been studied in phase 1 clinical trial. Actually, this strain is a genetically modified *Bifidobacterium longum* (*B. longum*) which was designed to combat against refractory solid tumor by delivering gene encoding the inflammatory factor interleukin-12 (IL-12). There is hope that bacTRL-IL-12 will be able to stimulate antitumor immune response. The size and volume of the tumor usually calculated after every cancer therapy were reduced after the use of the genetically engineered strains. This new way in the cancer therapy leads to both the targeted treatment of cancers and the development of antitumor compounds. The criteria in the designing of GEMs for cancer therapy include the ability to escape recognition by the host's immune system, invade the tumor, multiply in tumor cell, produce and release toxins or anticancer substances designed to carry into the tumor cell, and activate apoptotic genes in cancer cells [162].

### 9.2. Engineered Microorganisms for Inflammatory Disease

Inflammation is defined as complex biological processes and protective responses in the beginning of the innate immune response to antigens or microbes. However, inflammation is sometimes involved in the development of various diseases, e.g., neurodegenerative diseases, autoimmune diseases, and cardiovascular diseases [163, 219]. Inflammatory bowel disease (IBD) as a chronic inflammatory disease is characterized by recurrent and severe inflammatory responses including ulcerative colitis (UC), pouchitis, and Crohn's disease (CD) [165]. Leading cause of IBD has not yet been determined, but it is known that cytokines play an important role in the progression of the disease [167, 220]. The common treatment for IBD is a combination of anti-inflammatory and immunosuppressive therapies such as methotrexate, 5-aminosalicylic acid, corticosteroids, antitumor necrosis factor TNF-*α*, and surgical resection which in few cases may be ineffective or show side effects [165, 168, 170, 221]. In addition, current therapies for inflammatory diseases do not treat the origin of inflammation but relieve the symptoms; therefore, these treatments are not very effective, necessitating development of promising therapies [219].

Recently introduced treatments, such as fecal microbiota transplantation (FMT), probiotics, and prebiotics were reported as safe and more effective [171, 172]. Also, GEM is used as a new method in the treatment of many diseases especially inflammatory diseases [222]. Engineered *L. lactis* strains were designed by Steidler et al. to produce IL-10 as an anti-inflammatory cytokine. Results of their study showed 50% reduction in colitis symptoms in murine chronic colitis model induced by dextran sulfate sodium (DSS). In addition to being cost-effective, this treatment method also causes localized delivery and an active synthesis in situ. Besides, the dose required in this condition is lower than the dose required for systemic treatment. So, this strategy might represent a better way for the long-term and cost-effective management of IBD in patients [174, 175]. In a placebo-uncontrolled study, the *thymidylate synthase* gene of *L. lactis* was replaced by the *IL-10* gene to treat Crohn's disease patients. Treatment with this modified bacterium (LL-Thy12) alleviated the disease with the minimum side effects. It was demonstrated that employing genetically engineered bacteria for protein delivery to the mucosa is an appropriate approach in humans. As a result, it could be useful as a maintenance therapy for chronic intestinal illness [200]. Engineered *Bifidobacterium* spp., *B. longum*, also have been developed for the treatment of IBD to deliver *α*-melanocyte-stimulating hormone (*α*-MSH) exhibiting an anti-inflammatory effect on the intestine. Well intestinal colonization with engineered *B. longum* and significant anti-inflammatory effects by high *α*-MSH expression were observed in rat model of ulcerative colitis. Actually, this engineered bacterium reduces IL-6, TNF-*α*, nitric oxide (NO), and myeloperoxidase enzyme from which all are proinflammatory factors in ulcerative colitis and also increases IL-10 as an anti-inflammatory cytokine [174, 223]. Another strategy used in the treatment of colitis is the expression of enzymes with antioxidant properties. For instance, *Streptococcus thermophilus* (*S. thermophilus*) strains genetically modified by a plasmid to express catalase and superoxide dismutase were able to reduce colitis in a mouse model by reducing reactive oxygen species (ROS) production. So, these findings suggest that genetically altering a bacterium candidate with inherent immunomodulatory capabilities (e.g., *S. thermophilus* CRL 807) by inserting a gene encoding an antioxidant enzyme improves its anti-inflammatory effects to express catalase and superoxide dismutase and is able to reduce colitis in a murine model [177]. Neutralization of IL-6, as another important cytokine in the pathology of IBD, can be found as an effective treatment method. According to this statement, *L. lactis* strains expressing anti-IL-6 affibody has been engineered by fusion and expression of IL-6 on the surface of the probiotic with Usp45 (secretion peptide) and AcmA (anchoring protein). Findings of the investigation showed that this anti-IL-6 affibody can strongly remove human IL-6. The elimination was highly selective for IL-6, with no cross-reactivity for other proinflammatory cytokines linked to IBD pathogenesis. Because lactic acid bacteria survive in the gastrointestinal tract, they are appropriate for oral administration, enabling them for local delivery of cytokine blockers to the gut. Oral administration allows for direct contact with irritated mucosa, allowing medications to be delivered close to reactive cells. In IBD patients, structural abnormalities enhance bacterial buildup and medication transfer to the underlying lamina propria [224].

In addition to bacteria, bacteriophages can also be used in genetic engineering technology to express anti-inflammatory peptides on the surface of phages for the treatment of various diseases, e.g., inflammatory diseases [61]. The study showed that using genetic engineering to target the tumor necrosis factor-*α* receptor (TNFR1) can be a suitable option for the treatment of Crohn's disease induced by an increase in the expression of the inflammatory cytokine (TNF-*α*). Hydrostatin-SN1 (H-SN1) peptide in the T7 phage library obtained from the venom of the snake *Hydrophis cyanocinctus* (*H. cyanocinctus*) by cloning system exhibits its anti-inflammatory effects through targeting TNFR and preventing the binding of TNF-*α* to TNFR in a murine model, resulting in alleviation of colitis symptoms. It has been shown that in vitro, H-SN1 decreases TNF-*α* toxicity as well as the activation of TNFR-related signaling pathways such as mitogen-activated protein kinase (MAPK) and nuclear factor kappa light chain enhancer of activated B cells (NF-кB) [62]. Psoriasis is another example of inflammatory disease in which proliferation of skin cells and production of inflammatory mediators such as IL-1b, -6, -8, -17, -18, -23, and -36, CCL5, TNF-*α*, and interferon (IFN) *α*/*β* take place; the use of anti-inflammatory drugs and prevention of proliferation of keratinocytes are the current treatments for psoriasis [225–227]. Vazquez-Sanchez et al. investigated the anti-inflammatory activity of the heptapeptide HP3 expressed on the surface of phage in psoriasis animal models via phage display technology. Results of their study showed that HP3 inhibits the binding of peripheral blood mononuclear cells (PBMCs) to endothelial cells, leading to a reduction in PBMC migration and consequently a reduction in inflammation. Peptides represent a type of therapy with the advantages of low immunogenicity and high activity, and the phage display approach is a useful way for screening a wide range of therapeutic peptides with high selectivity and affinity, including anti-inflammation peptides [61, 226].

### 9.3. Engineered Microorganisms for Disorders

Metabolism of substances in the healthy person is relatively stable in a normal state but sometimes chemical processes due to abnormalities in the host's metabolism enzymes can lead to accumulation or even deficiency in these metabolites and consequently induction of disease [85, 134, 228, 229]. These diseases include autoimmune disorders such as arteriosclerosis, encephalitis, and metabolic disorders. Diabetes, cardiovascular disease, and obesity induced by changes in lifestyle, consuming unhealthy foods, etc. are the most common metabolic disorders [179, 180]. These diseases are difficult to treat and need long-term changes in diet and lifestyle patterns which impose a significant economic burden on patients [109]. Given that altered composition of the gut microbiome is another leading cause of these disorders, microbiome restoration is a helpful factor in their treatment. A new strategy for the modification of the gut microbiota could be use of modified microorganisms expressing and/or secreting therapeutic compounds [180]. In addition to targeted drug delivery, engineered bacteria as therapeutic agents can also help to restore homeostasis within a disturbed microbial population [230].

One of the applications of modified bacteria is in treatment of obesity which unfortunately has been increased significantly in the last 25 years. This chronic metabolic disorder considerably increases the risk of cardiovascular diseases and diabetes. In addition, since obesity is associated with inflammation, it may lead to autoimmune diseases such as IBD [230–232]. Surgery and drugs commonly are used for treatment of obesity, but due to the long-term impacts of the current diets and lifestyles, these treatments are not responsive [180, 233–236]. Transformation of *E. coli* by the N-acyltransferase gene obtained from *Arabidopsis thaliana* (*A. thaliana*) resulted in development of a new strain called At1g78690 to prevent the absorption of fats through different mechanisms such as oxidation of fatty acids and reduction of food consumption by expressing N-acylphosphatidylethanolamines (NAPEs) as precursors of N-acylethanolamide (NAE), leading to obesity control [180].

Another important disorder is uremia, which occurs following increase in uric acid levels resulting from the decrease in the excretion rate and subsequent accumulation in the kidney [237]. Uremia mostly is caused by renal failure, diabetes, and excessive consumption of alcohol. One approach for maintaining normal uric acid levels is oral administration of genetically engineered *E. coli* DH5 cells [238]. The urease gene isolated from *Klebsiella aerogenes* (*K. aerogenes*) was inserted to the *E. coli* DH5 strain. The *E. coli* strain was genetically modified to form a sodium alginate-encapsulated semipermeable membrane to persist throughout the gastrointestinal tract. Then, urea molecules quickly spread inside the microcapsules containing bacteria, resulting in reduction of urea amount. These modified bacteria orally were administered and completely excreted through the feces, indicating the safe use of this strain [239, 240].

Hyperglycemia is a typical symptom of diabetes which could be treated with live bacteria due to stimulation of intestinal epithelial cells to make insulin in response to glucose. The treatment of type I and type II diabetes became possible through the genetic modification of bacteria to improve production of insulin from host cells. Bacterial species were genetically engineered to generate insulinotropic proteins such as glucagon-like peptide-1 (GLP-1) and pancreatic *β*-cells-specific transcription factor pancreatic and duodenal homeobox 1 (PDX-1) [240]. Various species of genetically engineered bacteria including *E. coli* species and some probiotics showed promising results in reducing hyperglycemia and improving diabetes. For example, genetically engineered *E. coli* species can produce insulinotropic proteins including GLP-1 or pancreatic *β*-cells-specific transcription factor PDX-1 from pancreatic *β*-cells and intestinal cells. Similar studies used an engineered *Lactobacillus* to reduce hyperglycemia in mice through secreting GLP-1. Also, some bacteria especially *E. coli* Nissle strain increased glucose uptake by pancreatic endocrine cells with upregulation of Notch associated with the ngn3 gene by using expression GLP-1 or PDX-1 [132, 241–243].

The *phenylalanine lyase* gene was genetically heteroexpressed in *L. reuteri* 100-23C which was able to reduce the phenylalanine in murine model. Although oral administration of genetically modified probiotics was expected to be a good method for treatment of phenylketonuria (PKU), long host colonization by the probiotic was considered as a problem [244]. A similar study showed that insertion of the L-amino acid deaminase and phenylalanine lyase genes into the genome of *E. coli* Nissle 1917 to develop a novel engineered strain, SYNB1618, can solve the problem in treatment of PKU. PKU is an autosomal recessive disease in which a genetic defect in the phenylalanine hydroxylase leads to an increase in the blood phenylalanine, resulting in severe neurological complications including severe and irreversible mental disability, behavioral disorders of acquired epilepsy and microcephaly, seizures, psychological distress, and general hypopigmentation of the skin [120, 245]. Another study showed that the administration of *E. coli* Nissle 1917 in a murine model reduces the blood phenylalanine concentration by 38% through the expression of enzymes involved in degradation of this amino acid in mice [197].

Hyperammonemia as a kind of metabolic disorder occurs when there are extremely high levels of ammonia in the blood and requires immediate treatment. If untreated, hyperammonemia could be toxic and lead to coma or death. This complication can be the result of liver cell disorders or lack of urea cycle enzymes induced by the disturbance in ammonia clearance as a neurotoxic metabolite [85]. Kurtz et al. observed that oral administration of an engineered *E. coli* Nissle 1917 (SYNB1002), which can convert ammonia into L-arginine, leads to reduction of the ammonia amount and the survival of mice [135]. One of the main causes of death worldwide is alcoholic liver disease, which can be improved through genetically engineered bacteria such as *L. lactis* and *B. subtilis*. This innovative technique causes alcohol detoxification and reduces alcoholic liver damage through the expression of the alcohol dehydrogenase and aldehyde dehydrogenase genes in the genetically modified bacteria [246].

### 9.4. GEMs for Infections

Bacteria are undergoing genetically modifying technology based on their special characteristics. They can be designed to express specific biomacromolecules on their surface and are able to be used for the treatment of microbial infections [140, 240, 247]. Moreover, bacterial antibiotic resistance is increasing through excessive misuse of antibiotics which is directly related to an increase in death rate. Therefore, finding more effective alternative ways for treating infection and overcoming resistance is important [248, 249]. One of the strategies to combat infections using modified microorganisms is identifying internal and external QS signals secreted by pathogenic organisms and responding to them by the production of antimicrobial compounds and interference of QS mechanisms followed by suppression of virulence genes through alternative QS signals. QS refers to the bacterial interaction with one another or between species to coordinate cellular function [197, 250]. This process entails the identification of signaling molecules known as autoinducers (AIs). Spore formation, invasion of pathogens, bioluminescence, and population control are examples of QS-based phenotypic characteristics in bacteria [251]. Numerous studies were conducted to control the expression of pathogenic virulence genes and the production of antimicrobial compounds by analyzing biological systems and guiding their design based on QS. Commensal *E. coli* strains were genetically modified to recognize the signals of wild-type of *P. aeruginosa* (PAO1) and produce bacteriocin. This bacteriocin-producing probiotic expressing *Las*R was modified under the control of *lux*R promoter and E7 protein lysis to enhance the release of bacteriocin. *P. aeruginosa* growth and biofilm formation were reduced by 99% and 90%, respectively, when *P. aeruginosa* and recombinant *E. coli* were cultured [252, 253]. Mao et al. engineered *L. lactis* limiting the development of cholera in a mouse model. *V. cholera* produces two quorum sensing molecules including autoinducer-2 (AI-2) and cholera autoinducer 1 (CAI-1) in which CAI-1 is a signal for the expression of virulence genes; meanwhile, in low density of bacteria, the expression of virulence genes was increased. Through genetic engineering modification to express the receptor of CAI-1 on *L. lactis*, a high number of *V. cholerae* and a reduction in expression of virulence genes occurred, conferring protection in mice intestine from the progression of *V. cholera* [254, 255].

Another strategy can be destroying the membrane of bacteria. *Helicobacter pylori* (*H. pylori*) as Gram-negative bacteria cause gastrointestinal diseases and antibiotics are the only current therapy, while excessive use of antibiotics leads to an increase in antibiotics-resistant *H*. *pylori* strains. Xu et al. designed an engineered *E. coli* strain to express an artilysin which is composed of holin and endolysin. Holin as a protein that can make holes in the bacterial membrane in combination with endolysin which destroys the peptidoglycan amide bonds is produced by phages. The results indicate bacteriostatic impacts of artilysin on *Helicobacter* by perforation and destroying membrane [256]. Secretion of antibodies and adhesive subunits to prevent pathogen colonization can be useful in preventing infections. Toxins, secretion systems, and expression of pathogen adhesins may be promising targets for developing new anti-infective therapies for improving GEMs' ability to compete with pathogens [150]. *L. casei* was modified to express ETEC adhesion K99 (or K8872). Oral vaccination of modified *L. casei* resulted in high amounts of mucosal IgA in lung and gut fluids, high systemic IgG response in animal model study, and protection of more than 80% of mice against lethal dosage of the ETEC [257]. Finally, toxin neutralization through the modification of surface components, the generation of antibodies that neutralize these toxins, and the modification of genes encoding proteins or enzymes capable of neutralizing or breaking down toxins is one of the GEM antimicrobial strategies. For example, heat-sensitive receptors of ETEC and cholera toxin of host cells were cloned on the surface of the nonpathogenic probiotic *E. coli* by transferring glycosyltransferase genes from *Campylobacter jejuni* or *Neisseria meningitidis* to the *E. coli*. The results showed that engineered *E. coli* protects the host against diarrhea through the isolation of enterotoxins [258–260]. The engineered *E. coli* Nissle 1917 has been used against vancomycin-resistant *Enterococcus* (VRE) and its colonization in the intestine. Three peptides including Hiracin JM79, Enterocin A, and Enterocin B have been designed in *E. coli* to express and kill *Enterococcus* spp. These peptides are sufficiently produced and significantly prevent the growth of *Enterococcus* in vitro. Then, the effect of the modified probiotic was assessed in mice model colonized with *Enterococcus*. The results showed that the levels of both *E. faecium* and *E. faecalis* in the feces of mice have decreased significantly [261]. Since phages are promising therapeutic options due to their special features, such as the ability to be self-replicating and self-limiting, the usage of phage cocktails, and the possibility of modification of phages [262], phage engineering was introduced in genetic engineering by mutation, genetic replacement, and the integration of a foreign gene to increase the antibacterial spectrum effect of phage [249, 263]. Engineered *λ* phage inhibited the growth of Enterohemorrhagic *E. coli* (EHEC) both in vivo and in vitro. A CRISPR-Cas-3 system and several CRISPR spacers targeting EHEC are embedded in the wild-type *λ* phage. In fact, this CRISPR-Cas-3 system has been engineered to increase the specificity of this phage so that its lytic gene, *cro*, has been knocked out. The results of studying in mice model showed that GM *λ* phage reduces the number of bacteria to an undetectable level and not only rescues the mice but also restores the gut microbiota of the mice [264].

## 10. Routes of Administration for Engineered Microorganisms

The administration of genetically engineered microorganisms depends on factors such as the type of disease, the involved target tissue, and especially the pathogenic potential of the selected microorganism. Subcutaneous injection, intravenous, intratumoral, nasal, and oral administration methods have been defined for the transfer and administration of genetically engineered microorganisms [265]. Intravenous administration is exclusively utilized for transferring genetically engineered microorganisms in cancer treatment due to its high potential for systemic circulation [266]. In addition, intratumoral administration is also suggested for cancer therapy. In this method, the toxicity caused by the transfer of genetically engineered microorganisms is greatly reduced. These microorganisms multiply locally in the tumor site, so they stimulate the immune response, and the process of cell apoptosis is also started. However, this method faces probable complications such as inciting inflammation, cytokine storm, and the dangers of rapid cell death for other organs [267–269]. Nasal administration has been more successful in the administration of intranasal vaccines due to the induction of humoral and cellular immune responses both in the area of entry and the surrounding related areas [270]. In bacteriotherapy, oral administration is the most widely used administration method due to the fact that it is easier to perform and noninvasive, which leads to more acceptance by patients. However, the passage of genetically engineered microbes through the gastrointestinal system has always been criticized because the gastrointestinal system faces many challenges, such as diversity and differences in the microbial flora, the level of acidity (range of pH 1.0–7.4), and the percentage of oxygen (stomach, small intestine, and colon) [271–273]. During oral administration, approximately half of the genetically engineered microbes are lost due to the challenges mentioned earlier. Considering that these modified microbes need to be transferred intact and alive to the target tissue or organ in order to exert their optimal effects, loading them into carriers and covering them with lipid, polysaccharide, cationic nano-liposomes, and alginate helps protect them during the oral route of administration [27]. By comparison of these methods, blood and nasal administration of genetically engineered microbes may be more effective than oral due to their fast reaching to the target sites [269]. On the other hand, oral administration is much better than intranasal administration because it induces a wide immune response [274]. The difference in these results originates from the examination of different diseases, for example, allergic disease is more affected by oral administration than intravenous administration [275].

## 11. Influencing Parameters for the Effectiveness of GEMs

Based on the studies, the effectiveness of genetic engineering microbes both in the treatment and prevention of diseases as well as in the diagnosis of diseases depends on their dose of administration, the ability of colonization in the target area, and how much pathogenic potential they have (safety) [27]. The verification of colonization of genetically engineered microbes following administration to the body shows their survival during transit and reaching the target organ. Researchers use tools and techniques such as 16S sequencing, labeling with fluorescent dyes, molecular tests, and tissue sectioning. Genetically engineered microbes labeled with fluorescence can be discovered under fluorescence microscope in the feces of patients, or compared to the patient's feces before and after administration in terms of microbial flora [276]. Following the administration of modified microbes to the host body, the prescribed dose is considered the second crucial factor for effectiveness against diseases. For example, Whorwell et al. showed that *Bifidobacterium* was much more effective only in the number of 10^8^ cfu/ml for treatment of the irritable bowel syndrome (IBS) [277]. Therefore, in bacteriotherapy, the number of prescribed bacteria has a direct relationship with the significant effect that is expected. It seems that the more prescriptions of bacteria result in more effectiveness, but this is a misconception since an undetermined number of bacteria may cause side effects such as a cytokine storm [161]. For the purpose of reducing any possible toxic effects of genetically engineered microbes, before clinical application, they are subjected to chemical changes. Genetically engineered bacteria are more prone to induce complications; therefore, expression of main virulence genes was prevented or removed, like removing the gene encoding lipopolysaccharide in Gram-negative bacteria [278].

## 12. Safety Assessment

### 12.1. Assessment of Safety for Humans

According to research studies, various microorganisms including bacteria, viruses, and fungi have a high potential to treat diseases such as HIV infection, colitis, and cancer trough applying genetic changes to them. Engineered viruses can be used in treatment of cancer and hereditary vision loss due to retinal dystrophy and in prevention of alcoholism [279–281]. Various applications of engineered bacteria in different fields include combating specific pathogens, solving the emerging of antibiotic resistance, electrical conductivity, and maintenance of uric acid levels in treatment of obesity, as well as removal of pollutants in nature. Also, engineered bacteria undergo an apoptosis without remains [282]. Prevention of malaria transmission through restraining sporozoites from sticking to salivary glands is one of the applications of bioengineered fungi in the medical field [222, 283, 284].

One of the goals of modern medicine is targeted delivery of the maximum amount of drug in the target area to minimize off-target effects. To achieve this goal, four key components including delivery vehicle, sufficient stability in the target site, retention, and timely release of the medicine are needed. Since systemic administration of drugs does not have some of these features, they cause many challenges in treatment. Considering GMOs' positive effects, interest in using new techniques such as genetic engineering, nanoparticles, and biopolymers recently has increased [179, 285, 286]. Although this novel treatment method overcomes obstacles of traditional drug treatment such as side effects, effective delivery, cost, and dosage, several technical hurdles described for them should be considered before their use [179, 287]. One of these obstacles is the adverse effect on competition with normal flora for niches in the intestinal environment, which is induced by the metabolic burden of recombinant genes in engineered microbes. Also, genetically modified bacteria carrying antibiotic resistance genes and plasmids and causing horizontal transfer of genes are not clinically ideal. Another obstacle is about integration tools, which are not available for all strains and species, while the chromosomal integration usually provides a safer and more stable method for engineering. Furthermore, GM bacteria contain plasmids carrying antibiotic-resistance genes that allow them to horizontal gene transfer, resulting in subsequent genetic modifications in bacteria through the spread of antibiotic-resistance genes that are not ideal. Another obstacle is the lack of available integration tools for all strains and species. However, chromosomal integration of the expression cassette without antibiotic selection markers usually provides a safer and more stable method of engineering [288, 289].

In contrast to other small molecules, live bacteria and bacterial spores cannot be sterilized using heating and filtering methods, which is considered an important challenge in their production, so the final products must be examined for pathogenic agents or pathological conditions before consumption [278]. The effective dose is not necessarily associated to the administered dose; mostly it depends on the target tissue, which is challengeable. Since live bacteria (both harmful and therapeutic) have different tendency for colonization of target tissue and subsequent multiplication, their dose may not be effective especially in tumor tissues [211, 290]. In addition to toxicity, control of bacterial colonization, potential biosafety and biocompatibility, bacterial viability during delivery, long-term safety, and living biological agents in a clinical environment are serious concerns due to their potential impact on the environment and public health, introducing new challenges for using this technique [140, 179, 230].

### 12.2. Assessment of Safety for the Environment

The usage and release of GEMs into the environment, typically agriculture, is expanding exponentially. GEMs' potential uses include mining and mineral recovery, crop production, and insect management [28]. This technique can also favor other components of agricultural emissions, such as lowering the use of energy and fossil fuels and permitting decreased tillage and no-tillage farming methods, in addition to decreasing emissions from changing land cover and early pesticide usage [291]. Furthermore, it might be a powerful tool for the broader conservation of biodiversity. According to projections, between 15 and 40 percent of species might become extinct by 2050, mostly due to habitat loss and altered conditions brought on by climate change, so it requires more attention in this field [30]. In the recent era, the increased persistence of hazardous contaminants is having a negative impact on the world in a variety of ways [31]. Compared to other physicochemical approaches, utilizing microbial metabolic abilities for the degradation/removal of environmental contaminants provides an economical and safe alternative [32]. Despite the many advantages of GEMs in bioremediation and despite many regulations to prevent them from harming the environment, environmental concerns and legal issues related to the release/use of GEMs have limited their use [33–35]. It is very challenging to define the environmental risk associated with the intentional release of GEMs in a clear and concise manner. Several definitions have been put forth; the term “ecosystem perturbation” may be the most nebulous since it can refer to almost any possible outcome (e.g., disturbance of ecological processes such as soil respiration, nitrification, or denitrification) [36]. One of the environmental concerns with GMOs is the potential spread of introduced traits to wild populations. This could occur through cross-pollination of genetically engineered (GE) crops with wild relatives, or through horizontal gene transfer (HGT) mechanisms. The former is very unlikely in most cases because commercially grown crops are usually genetically distant from their wild neighbors, but it has been observed. Some current regulations and practices aim to reduce the frequency of these occurrences [292]. The latter appears to be extremely rare in eukaryotes, according to Keese [38]. It is shown in this regard that many species of microalgae studied for biofuel production can invade ecosystems due to their small size, rapid growth, and large numbers, causing environmental concerns such as competition, habitat alteration, horizontal gene transfer, and toxicity. Among other major concerns about the spread of GEMs use are the risks related to the persistence of unwanted genes, the transfer of GEMs to native species, and uncontrolled propagation, especially when they are to be widely distributed [39, 293]. The serious consequences of vertical gene transfer between GMOs and their wild-type counterparts were shown in studies involving transgenic fish released into natural populations of the same species. The genetically altered fish's enhanced capacity for reproduction led to a reduction in the survivability of their offspring [40]. Furthermore, to prevent the introduction of unwanted genetic features and make the behavior of a GEM more predictable, the capacity of GEMs to disseminate new genetic information to potential recipients must be restricted. Also, in order to prevent GEMs from spreading and having an adverse effect on the native population of organisms, their survival must be restricted in both time and space, or through the establishment of a controlled life cycle (biological confinement) [39]. For instance, during a study, it was stated that the growth of monarch butterflies by consuming pollinated leaves from genetically modified corn is slowed down and the chance of their death increases because GM crops reduce the number of insects that provide food for birds and other wildlife, and from this it harms biodiversity [41]. Genetically modified foods and the spread of antibiotic-resistant genes to gut flora can expose people to new allergies [40]. For example, Seralini et al.'s study revealed that rats fed transgenic NK-603 Roundup Ready maize had higher tumor incidence, chronic kidney disease, liver congestion, necrosis, and higher female mortality [42]. In addition to putting human health at risk, horizontal gene transfer of pesticide, herbicide, or antibiotic resistance to other species will upset ecological balances by enabling previously innocuous plants to proliferate unrestrained and so boosting the spread of pathogens between plants and animals [43]. Additionally, recent research has demonstrated that probiotics can evolve undesirable traits during treatment or diagnosis including acquiring harmful functions such as competitive elimination of native microbes, pathogenic potential against host or environment, or loss of beneficial functions of the engineered system; therefore, biocontrol strategies preventing or minimizing the entry or any penetration of GMOs into the environment are needed [44, 45, 260, 294–296]. Although we may not be able to accurately predict the interaction of each genetically engineered microorganism with the environment, several biocontainment strategies of various degrees of efficiency and stability have been developed such as the use of auxotrophy and synthetic amino acids. Auxotrophy is the elimination of the ability of an organism to synthesize a vital compound that it must receive from its growth medium or its environment and can help to inhibit microorganism in the laboratory environment. These vital compounds include somethings essential for cell survival or those needed in high concentrations which cause the maximum effectiveness of this method [250, 297–299]. Notably, inducible systems or auxotrophy does not address the potential risk of genetic information escape, i.e., horizontal gene transfer. Moreover, the limitation of this method is that they may require additional survival factors and supplemental probiotic media [197, 251]. Cellular circuits contain lethal switches, as well as addiction modules, all of which achieve inhibition by binding cells to a specific compound (or lack thereof) or genetic information, and thus lethal components are expressed in response to environmental signals and finally cause cell death in response to an inducing chemical. Control of cell survival is done through expression of lysis proteins and destruction of essential proteins and toxins. For example, in *E. coli,* the “bioretention circuits” included temperature sensors to differentiate between physiological and environmental temperatures [150, 251–253, 300, 301]. Another solution for intoxicating GMOs in environment is toxin-antitoxin systems consisting of stable and unstable toxins. To prevent the transfer of genetic information in the horizontal gene transfer, toxin and antitoxin systems can be placed in different parts of the cell's genetic repository. For example, the toxin is placed on the plasmid and the antitoxin on the chromosome; therefore, during horizontal gene transfer, the plasmid that does not carry the antitoxin will kill the new host [257]. Understanding how to evaluate the impact on biodiversity is necessary for this technology to be accepted despite its many positive effects on the environment and economy. The assessment of future genetically modified organisms' effects on biodiversity requires ongoing updates to this knowledge (https://www.government.nl/topics/biotechnology/consequences-of-gmos-for-biodiversity). Due to their inherent properties in degradability, they are eliminated by themselves in the body and will not have side effects or will be less. However, their safety and control still need to be studied (2).

## 13. Assessment of Stability of Genetic Construct

The delivery of drugs using genetically modified organisms (GMOs) and their utilization in treatment offer several advantages over traditional treatments. These advantages include a significant reduction in costs through the production of a large number of engineered bacteria compared to biological molecules. Furthermore, treatment with GMOs can result in limited side effects due to their nonaggressive nature during administration. Also, less dosage than biological compounds is another advantage of GMOs, but before using these strains for clinical trials and recommending them to humans, considerations such as genetic stability, avoiding antibiotic-resistant genes, and maintaining genetic integrity are necessary to produce them in high concentrations [197, 258, 259]. Use of regulators and adding sensors, kill switches, delivery tools, and memory circuits have paved the way for genetic engineering. To increase genetic stability, most GMOs carry recombinant DNA in their chromosomes because plasmids are not genetically stable due to unequal distribution between daughter cells during cell division; also, they are lost in the absence of selective pressure [109, 120, 196, 302]. In addition, by removing an essential gene and using a single copy of plasmid from the same gene in the host, the induction and suppression systems can be used for long-term maintenance of plasmids and to overcome the genetic stability challenge. *E. coli* strain Nissle 1917 designed in Danino et al.'s study to harbor the toxin and the alp7AR cassette encoded in the short-lived plasmid provides equal separation during cell division by producing alp protein strands and the antitoxin encoded in the host chromosome, which ultimately causes cell death when this plasmid is transferred [109, 196].

### 13.1. The Effectiveness and Safety of GEMs in Targeted Drug Delivery

Genetically modified bacteria purposefully have properties such as destroying biofilm and reducing resistance to antibiotics and being degradable. As recent studies have pointed to the effective role of target delivery of GEMs [303] and carrying the drug, GEMs can directly deliver the drug to the desired area [304]. In addition to nanoparticles (NPs), synthetic biology has provided the possibility of changing bacteria for specific applications such as vectors for inoculating nucleic acids into cells. The genetics of these microorganisms are modified by genetic engineering so that they have the least toxicity and have the greatest effect in the targeted transfer to the desired tissue [303].

When bacteria reach the body, depending on what purpose and for what disease they are designed, they must access the target tissue or cells and then multiply quickly in the target area. Tumor cells produce compounds that act as chemotaxis for bacteria and direct them to the target cell. Areas with low oxygen concentration are suitable places for inherently anaerobic bacteria such as *Clostridium* and *Bifidobacterium*, which were designed to be effective in these areas [305]. Effective transmission to the target in the case of *Listeria* is mediated by exploiting the immune system. With this ability, they can infect antigen-presenting cells and myeloid-derived suppressor cells (MDSCs) and reach the target tissue using them like taxicab. In this way, these bacteria survive against the immune system [306, 307]. In addition, motility of mobile bacteria with flagella enables them to easily penetrate deeper points and swim to the desired points and spread [308, 309]. For enhancement of tumor targeting, genetically engineered strains are designed in ways that, in addition to antitumor properties, target accurately and have safety. An example of this is the ppGpp-deficient strain SHJ2037, which is designed by using genetic engineering to express tumor-specific ligand, an Arg-Gly-Asp peptide, which has high specificity to *α*v*β*3 integrin and can exert its anticancer effects on breast. Apply cancer cells and melanoma xenografts overexpressing*α*v*β*3 integrin [306].

In order to reduce possible off-targeting effects, the strains are designed to target only specific genes expressed on the cell or tissue, thus preventing the accumulation of modified strains in other organs and the occurrence of nonspecific complications [310, 311]. Expression of synthetically designed adhesins contributes to selectivity and increased affinity and specificity [152]. In addition, coating the cells with plasmid-loaded nanoparticles that can express a gene called bioluminescence helps to detect the engineered strain by bioluminescence when it reaches the target organ [106, 312].

Off-target effects are the unwanted side effects of the target sequence. Since CRISPR-Cas9 is known as one of the most common techniques in genetic engineering, it can be considered as one of the most important goals of reducing off-target effects to synthesize less dangerous strains. Unwanted genetic modification is a biosafety issue associated with the use of CRISPR-Cas9. In order to reduce off-target effects caused by CRISPR-Cas9, strategies such as Strategic gRNA Design that identifies only a special sequence, Truncated gRNAs, Cas9 Paired Nickases and “Enhanced specificity” SpCas9, or eSpCas9 can be considered [303].

When the bacterium enters the body, due to the complexity of the human body, the entry of the modified organism may be disrupted, and that is why it is very necessary to design a genetically modified strain that can adjust to release the drug at the right time. It actually goes back to studying the field of gene expression regulation. Following the identification and detection of a series of signals, the bacteria activate the downstream genes and start releasing the desired drug. Computing processes were designed based on temporal logic or linear time, which explains the timeline in any field. This is the link to the input signal. Scientists tried to introduce this at the genetic level, so by using synthetic biology and introducing the Feynman gate idea, they inserted a network into the bacteria and examined it. They defined isopropyl *β*-d-1-thiogalactopyranoside (IPTG) and anhydrotetracycline (aTc) as the input signal and fluorescence proteins EGFP and E2Crimson as the main output [304]. The obtained results fully confirmed the idea of Feynman gate. Therefore, when the genetic circuits are designed correctly, it leads to the genetically engineered strains playing a more consistent role in carrying the drug and releasing it, having less toxicity and leading to greater efficacy. In another example, *Escherichia coli* Nissle 1917 researchers designed synthetic gene circuits that regulate its encapsulation [305]. Therefore, this system that controls the level of bacteria leads to the regulation of immunogenicity, vitality, and drug load in bacteria, which is not only effective in improving effectiveness but also plays a role in improving safety. Depending on the concentration of IPTG inducer, this capsular polysaccharide (CAP) expression system regulates the biosynthesis of CAP genes. Additional studies showed that this system helps to maintain the life of the bacteria so that it is not cleared by the immune system and the desired area in the tissue will be removed after the completion of the treatment without leaving any side effects, and in synergy with other drugs that are usually used for treatment, they will lead to the reduction and clearing of tumors.

### 13.2. Limitation and Strength

The present study has not investigated GM fungi and other nonphage GM viruses, as well as the impact of GEMs on climate and animals. There are two reasons for this. First, the most widely investigated natural microorganisms that have been extensively studied in the past and their efficacy were recorded and discussed. Second, in the search to determine the effect of genetically engineered microorganisms (GEMs) on animals, no relevant studies were found. This review is comprehensive due to the complete study on types of genetically engineered bacteria and phages, therapeutic and diagnostic application of GEMs on disease, cancer, and metabolic and inflammatory disorders, and how manufacturing of GEMs, route of administration, and GEMs affect the environment. In future studies, it is suggested to investigate the effects of GEMs on climates and animals.

## 14. Conclusion and Future Perspective

There is progress in sequencing, synthetic biology approaches, genetic engineering, and understanding pathways such as the gut-brain axis aimed to clarify the relationship between microbes and humans and the development of genetically engineered bacteria and phages. The in vitro and in vivo studies investigated the effect of GEMs as therapeutic, prophylaxis, diagnostic, and delivery strategies for diseases, disorders, and cancer and indicated GEMs have low cost, high efficacy, and no side effects, even more effective than conventional therapies in cases. GEMs are effective by competing for adhesion to main receptors, targeting tumor cells and replication in them, and carrying antimicrobial proteins in antibiotic-resistant diseases. However, GEMs are still in their infancy and face limitations. There is a need to investigate the impacts of GEM on the environment and climate, genetic stability, and safety usage of them. In the future, GEMs are expected as promising complementary and adjunctive therapy to improve human health.

## Figures and Tables

**Table 1:** Studies on therapeutic effects of genetically modified phages.

Engineered phage	Method of making engineering phage/route of ad	Target	Mode of action of engineered phage	Outcome	Ref
M13mp18	Insert genes in pZE11G vector and cloned in phage/IP	*E. coli* K-12	Express *lexA3*, *soxR, csrA,* and *ompF* genes on phage	Enhance penetration and bactericidal activity of antibiotic	[70]

T7	Integration genes in phage by homologous recombination in *E. coli* host strain	*E. coli* DH5a	Expression *lnqQ* and *trxA* on T7 phage	Production of bacteriocin Increased T7 phage lytic activity which leads to prevent the emergence of resistant bacteria	[71]

M13	Phagemid constructed by cloning by plug-and-play cloning platform/IP	*E. coli*	Expressing antimicrobial peptides (cecropin, apidaecin) and protein toxin (ccdB) on phagemid	Interfere with bacterial intracellular processes such as septum formation, DNA replication, DnaK activity, and protein production	[72]

phi11	Cloning and allelic exchange techniques	*S. aureus*	Expression of *SASP* gene of *B. megaterium* on phage	Inactivation of DNA, enhancement of bactericidal activity, effective against all the *S. aureus* Low propensity for resistance development	[73]
M13	Phagemid constructed by cloning	*E. coli* O157: H7	Engineered a modified nonreplicating M13-derived phage expressing a lethal catabolite gene CAP	Killing of adenyl cyclase positive bacteria by lethal cap protein transfer	[74]

*λ*	Plasmid vector, inherent red recombination system/oral	*E. coli* MG1655	Phage that targets SXT2 toxin expression	In vitro lysogenize and suppress Stx2 synthesis in *E. coli* Drastic reduction of Stx2 production in vivo	[75]
NM1	Phagemid constructed by cloning by tracrRNA and CRISPR array/topically	*S. aureus*	Plasmid targets the *aph-3-*kanamycin resistance gene	Inactivation of target bacterial functions and immunization of nonvirulent strains against plasmid-borne horizontal transfer	[76]

M13	CRISPRCas13a/administration of phage into larvae	*E. coli* and *S. aureus*	Construct targeting antibiotic resistance genes (bla, mcr, mecA)	Killing activity against bacteria carrying the blaIMP-1 gene	[76]

T7	Insertion a construct into the phage genome with a T7Select 415-1 kit	*E. coli* BL21	Expressing of fluorescent marker mCherry and antimicrobial peptide	Eliminating both biofilms and planktonic cells	[77]

M13	*λ*-red recombineering using pSIM9 system	*E. coli* EMG2	Engineered a phage harboring RNA-guided nuclease that targets *bla*SHV-18 or *bla*NDM-1 and *gyrA* genes to break DNA in antibiotic resistance genes	Decrease in viable cells and DNA damage in target cells	[78]

M13	Phagemid constructed by cloning	*E. coli*	Expression of *λS105* holin in phage M13S105 and *BglII* restriction endonuclease in M13R phage	Decrease by more than 99% in bacterial viability	[79]

M13	Phagemid constructed by cloning/IP	*E. coli*	Expression of lethal agents Gef and ChpBK	Decreased target bacterial populations	[80]
T7	Cloning extracellular matrix polymer gene of *A. actinomycetemcomitans* in phage	*E. coli* TG1	Express DspB (dispersin B) intracellularly during infection	Efficient deletion of biofilm-producing bacteria	[81]

*λ*	CRISPR-Cas9	*E. coli*	Phage targets ndm-1 and ctx-M-15 genes (*β* lactamases encoding genes)	Loss of resistance determinants, minimization of horizontal transmission, instead of killing resistant bacteria, it sensitizes them and then enriches this sensitive population	[82]

*φ*SaBov	CRISPR/Cas9/SC	*S. aureus*	Deliver a CRISPR/Cas 9 carrying phage that targets *nuc* gene	Improve efficiency of *S. aureus*specific-killing by SaBov-Cas9-nuc to full eradication, and CFU reduction	[83]

M13 phage/*S. aureus* phage 80*α*	CRISPR-Cas13a/Administration of phage into larvae	*E. coli* NEB5- and *S. aureus* USA300	Phage that carries CRISPR system targets carbapenem-resistant genes (*bla*IMP-1, *bla*OXA-48*, bla*VIM-2, *bla*NDM-1, and *bla*KPC-2) and colistin-resistant genes (*mcr*-1 and *mcr*-2) in *E. coli*/CRISPRCas13a system in phage targets *S. aureus rps*E genes and the methicillin-resistant gene *mec*A	Specific killing of carbapenem and colistin-resistant *E. coli* and MRSA	[76]

M13	CRISPR-Cas9 phagemid vectors/oral	Streptomycin-resistant *E. coli*	M13, which carries CRISPR-Cas9, targets *E. coli* that has sfGFP (green fluorescence) marker gene	Strain-specific reduction of fluorescently marked isogenic strains during competitive colonization	[84]

SASP, small acid soluble spore protein; CAP, catabolite gene activator protein; tracrRNA, trans-activating crRNA; SC, subcutaneous injection; MRSA, methicillin-resistant *Staphylococcus aureus*; A.a, *Aggregatibacter actinomycetemcomitans*.

**Table 2:** Therapeutic effects of genetically modified bacteria including probiotic strains and other bacterial species.

Engineered microorganisms	Transformation tools (vector)/route of ad	Target	Method	Outcome	Ref
*L. lactis*	Plasmid-encoded genes/oral	*V. cholerae*	Engineered a strain to express the CAI-1	Inhibition of cholera progression and protection in high-risk populations	[115]
*L. reuteri*	Electrotransformation of plasmid-encoded genes	*S. aureus*	Engineered *L. reuteri* to sense AIP-I	Well detection of AIP-I levels and also *S. aureus* by recombinant *L. reuteri*	[116]
*E. coli Nissle* 1917	NA/oral	VRE	Developed an ECN to deliver and produce bacteriocins for killing VRE strains	Reduction of *Enterococcus* species in intestine	[117]
*Saccharomyces boulardii*	Plasmid-encoded genes transformation/oral	Antibiotic-resistant *C. difficile*	Engineered a strain to produce antibody to inactivate toxins	Protection against CDI	[118]
*L. casei*	Electrotransformation of plasmid-encoded genes	*L. monocytogenes*	Engineered *L. casei* to express the LAP from *L. innocua*	Prevent infection by competitive adhesion	[119]
*E. coli Nissle* 1917	Electrotransformation of plasmid-encoded genes/oral	*P. aeruginosa*	Developed a modified ECN to sense *P. aeruginosa* and kill	Protection animal model against *P. aeruginosa* during infection	[120]
*E. coli* strain “SLIC”	Electrotransformation of plasmid-encoded genes/IT and IV	Mouse CRC cell line (CT26)/mouse B-cell lymphoma line	Engineered a probiotic for release of nanobodies targeting PD-L1 and CTLA-4	Enhancement in tumor treatment and inhibition of tumor cell proliferation	[121]
*E. coli Nissle* 1917	Heat shock transformation/IV	Stage IV human breast cancer (4T1 tumor)	Engineered NIR light responsive strain as antitumor factor which is based on light responsive nanoparticles	Efficient tumor growth inhibition by well production of TNF*α* in tumor by NIR light responsive strain	[122]
*E. coli* Nissle 1917	Electrotransformation of plasmid-encoded genes/IV	Human hepatocellular carcinoma	Design to deliver p53 and Tum-5 proteins to tumor hypoxic area	Growth inhibition of the human hepatoma SMMC-7721 cells and no side effects on animal model	[123]
*E. coli* Nissle 1917	NA/SC	Mice colon adenocarcinoma	Developed an engineered probiotic to convert ammonia to an antitumor L-arginine	Increase level of L-arginine in tumor region which yielded by conversion of ammonia and significant effect on clearance of tumors	[124]
*L. lactis*	Electrotransformation of plasmid-encoded genes/NAS	Cervical cancer	*L. lactis* strain engineered to express IL-12	Production of IL-12 by *L. lactis* and enhancement of immune response	[125]
*B. longum*	Electrotransformation of plasmid-encoded genes/intragastric	Colon carcinoma cells	*B. longum* designed to deliver tumstatin (tum) into the solid tumor	Significant antitumor effects	[126]
*B. longum* 105-A	Electrotransformation of plasmid-encoded genes/IV	Lewis lung cancer	*B. longum* engineered to deliver spectinomycin-resistant gene	Successful gene delivery by *B. longum*	[127]
*Lactobacillus reuteri* 100-23C	Electrotransformation of plasmid-encoded genes/IP	PKU	Phenylalanine lyase gene cloned into a shuttle vector for expression in *L. reuteri*	Blood phenylalanine levels in PKU mice model significantly reduced	[128]
*E. coli Nissle* 1917	Electrotransformation of plasmid-encoded genes/oral	Colitis	Engineered ECN to produce the 3HB in the mice gut	Improvement in the colon characteristics like weight and lengths and proinflammatory cytokines of the gut tissue	[129]
*E. coli Nissle* 1917	NA	Ulcerative colitis	Developed an EcN as a nanosystem for diagnosis of UC at home based on production of IL-10	Detection of UC in less than 1 minute	[130]
*E. coli Nissle* 1917	NA/oral	Obesity	Genetically modified ECN effects on obesity	Well regulation and good effects on obesity	[131]
*L. gasseri*	Electrotransformation of plasmid-encoded genes/oral	Diabetes	Engineered *L. gasseri* to produce GLP-1 which is needed for changing cells to insulin-producing cells	Sufficient development of insulin-producing cells in the intestine following treatment by *L. gasseri* secreting GLP-1	[132]
*E. coli* Nissle 1917 (SYNB1618)	Electrotransformation of plasmid-encoded genes/	PKU	The genes encoding phenylalanine ammonia lyase and L-amino acid deaminase inserted into the genome of SYNB1618	SYNB1618 strains had transient colonies in the intestine and consumed phenylalanine in GI They were safe and tolerable	[133]
*E. coli* strain (SYNB8802)	Electrotransformation of plasmid-encoded genes/	Primary hyperoxaluria type I	Designed a strain to have ability to hydrolyse oxalic acid in GI	Safety and successful result of treatment Kidney damage reduction caused by hyperoxaluria	[134]
*E. coli* strain (SYNB1020)	Electrotransformation of plasmid-encoded genes/oral	Hyperammonemia	Design to hydrolyse ammonia into L-arginine	Improvement in the survival of mice	[135]
*E. coli Nissle* 1917	Electrotransformation of plasmid-encoded genes/oral	IBD	Designed sulfate sensor (ThsSR) and tetrathionate sensor (TtrSR) and transferred them into the ECN	Successful in diagnostics and therapeutics purposes	[136]
*E. coli NGF-1*	Construct was transformed by transduction/oral	IBD	Engineered *E. coli NGF-1* to sense tetrathionate	Well detection of tetrathionate	[137]
*E. coli Nissle* 1917	Electrotransformation of plasmid-encoded genes	IBD	Engineered an ECN strain to accumulate in desired sites and secrete GM-CSF in presence of NO	Successful movements of bacteria to NO	[138]
*E. coli Nissle* 1917	Electrotransformation of plasmid-encoded genes/oral	IBD	Designed EcN to produce curli-fused TFFs as fibrous matrices for strength and gut epithelial integrity	EcN produced the curli-fused TFFs and protection against colitis in animal model	[139]
*E. coli Nissle* 1917 (EcN-Sj16)	Electrotransformation of plasmid-encoded genes/IP	IBD	Designed EcN-Sj16 to express Sj16	Increase in expression of Sj16 in the GI and improvement in symptoms	[140]
*Yeast* strain BS016	Lithium acetate transformation method/oral	IBD	Designed an engineered yeast to express P2Y2 receptor to sense inflammatory	Inhibition of inflammation induced in IBD	[141]
*E. coli Nissle* 1917	NA/oral	IBD	Developed a PDNI coating ECN to regulate immune responses and improve gut microflora	Inhibition of excessive immune response and amelioration of colitis symptoms	[1]
*L. reuteri*	Electrotransformation of plasmid-encoded genes	*Staphylococcus aureus*	Development of a sensor to detect AIP-I and staphylococcal detection	Well and successful detection of AIP-I levels by engineered biosensor	[116]
*E. coli* Nissle 1917	Plasmid-encoded genes	*Pseudomonas aeruginosa*	Development of an engineered strain to detect the AHL and kill pathogen	Significant antibacterial activity	[120]
Harmless *E. coli*	Plasmid-encoded genes/oral	Pathogenic infection (enterotoxigenic *E. coli*)	Synthesis of a heat-labile enterotoxin-binding chimeric LPS with high avidity	Prevent and control diarrhea caused by enterotoxigenic *Escherichia coli* strains	[142]
*E. coli*	Plasmid-encoded genes	*V. cholerae*	Engineered a strain to express the Art-085	Inhibit the growth of *V. cholerae* by integrated sense and kill system in engineered strain	[143]
*B.subtilis*	Electrotransformation of plasmid-encoded genes/intragastric	Alcoholic liver	Coexpressing scADH and istALDH in food-grade *B. subtilis*	Alcohol detoxification and liver injury treatment	[144]
*B. ovatus*	Plasmid-encoded genes/oral	IBD	Modified *B. ovatus* for controlled in situ TGF-1 release and colitis treatment	Improvement in colitis, repair of injured colonic epithelium, decrease in inflammatory cell infiltration, reduction in proinflammatory cytokine expression, stimulation of mucin-rich goblet cell production in colonic crypts	[145]
*E. coli*	Transduction/oral	IBD	Development of an engineered strain to detect tetrathionate, which is produced during inflammation	Tetrathionate detection	[137]
*S. typhimurium*	Plasmid-encoded genes/oral	Cancer	Designed and engineered a clinical bacteria to lyse and release genetic cargo at a threshold population density	Significantly reduced tumor activity by combining chemotherapy and engineering bacteria	[146]
*S. Typhimurium*	N/A/IV	Cancer	Designed the Lux QS system and GFP reporter and transferred them into nonpathogenic *Salmonella*	Potential way to treat cancer by minimizing off-target therapeutic delivery	[147]
*S. typhimurium*	Electrotransformation of plasmid-encoded genes/intratumoral	Cancer	Stimulation of immune response in tumor tissue by induction of FlaB	Reduced tumor formation and metastasis in animal experiments while increasing survival time	[148]
*E. coli*	Transformation of plasmid-encoded genes/oral	Colitis	Designing invasive and nonpathogenic *E. coli* (dap) auxotroph, harboring plasmid pGB2Oinv-hly	Reducing the severity of experimental colitis in mice	[149]
*E. coli*	Plasmid-encoded genes/oral	Fever	Use from integration of molecular bioswitches into thermal logic circuits in three in vivo microbial therapeutic scenarios	Detect fever by thermosensitive promoters	[150]
*E. coli*	Electroporation of plasmid-encoded genes	Inflammation and glycosuria	Development of digitization, thresholding circuit, and amplification of nitrogen oxides and glucose in clinical samples	Detection of abnormal glycosuria in diabetes patients' urine using bactosensors	[151]
*E. coli*	Plasmid-encoded genes/IV	Cancer	Designed tumor-specific modular synthetic adhesins to improve targeting	Engineered bacteria with SAs colonize solid tumors with higher efficiency than the wild type of strain	[152]
*S. typhimurium*	Electrotransformation of plasmid-encoded genes/subcutaneous	Cancer	Design of an attenuated strain for IFN-*γ* secretion and expression	Treatment of melanoma	[153]
*B. subtilis*	Plasmid-encoded genes/oral	*H. pylori*	Design of *H. pylori* urease B on the *Bacillus subtilis* spore coat	Inhibition of *H. pylori* infection and development of an oral vaccine	[154]
*S. typhimurium*	Electroporation of plasmid-encoded genes/oral	Lyme disease	*S. typhimurium* strain engineered to expresses OspA	Protected against an intradermal challenge with the spirochete high titers of anti-OspA antibodies	[155]
*Streptococcus*	Plasmid-encoded genes	HIV	The production of an antiviral protein to prevent HIV infection in the vaginal mucosa	Maintaining an effective concentration of a microbicide in the vaginal mucosa by CV-N, an anti-HIV protein found in *S. gordonii*	[156]
*E. coli*	Transformation of plasmid-encoded genes/oral	*V. cholerae*	The genes encoding glycosyltransferase from *Neisseria gonorrhoeae* and *Campylobacter* inserted into harmless *Escherichia coli* strain	Toxin-binding probiotics have significant potential for cholera prevention and therapy in humans	[157]
*Salmonella*	Plasmid-encoded genes/intratumoral	Cancer	Designed a strain to have ability to delivery and production of Cp53 peptide	Death of tumor cells	[158]

ad, administration; EcN, *E. coli* Nissle 1917; PD-L1, programmed cell death-ligand 1; CTLA-4, cytotoxic T lymphocyte-associated protein-4; CAI-1, cholera autoinducer 1; AIP-I, autoinducer peptide-I; AHL, autoinducer N-acyl homoserine lactone; IT, intratumoral; IV, intravenous; IP, intraperitoneal; NAS, intranasal; CRC, colorectal cancer; PKU, phenylketonuria; CDI, *C. difficile* infection; NIR; near-infrared; 3HB, 3-hydroxybutyrate; IBD, inflammatory bowel disease; TFFs, trefoil factors; Sj16, schistosome immunoregulatory protein; PDNI, polydopamine nanoparticular immunosuppressant; LAP, *Listeria* adhesion protein; AIP-I, autoinducer peptide-I; VRE, vancomycin-resistant *Enterococcus*; UC, ulcerative colitis; LPS, lipopolysaccharide; Art-085, killing protein; *B. subtilis*, *Bacillus subtilis*; scADH, alcohol dehydrogenase from *Saccharomyces cerevisiae*; istALDH; aldehyde dehydrogenase from *Issatchenkia terricola*; *B. ovatus*, *Bacteroides ovatus*; QS, quorum sensing; dap, diaminopimelate; SAs, synthetic adhesins; IFN-*γ*, interferon-gamma; *H. pylori*, *Helicobacter pylori*; OspA, outer surface protein; *S. typhimurium*, *Salmonella typhimurium*; *P. aeruginosa*, *Pseudomonas aeruginosa*.

**Table 3:** Genetically modified microorganisms in different clinical trial stages.

Condition	Engineered microorganisms	Target	Clinical trial code	Clinical trial phase	Status
Metabolic diseases	*E. coli* (SYNB1934 and SYNB1618)	Phenylketonuria	NCT04984525	Phase 1	Ongoing
Metabolic diseases	*E. coli* (SYNB8802)	Enteric hyperoxaluria	NCT04629170	Phase 1	Ongoing
Cancer	*E. coli* (SYNB1891)	Metastatic solid Neoplasm and Lymphoma	NCT04167137	Phase 1	Ongoing
Cancer	*B. longum* (bacTRL-IL-12)	Solid tumors	NCT04025307	Phase 1	Ongoing
Metabolic diseases	*E. coli* (SYNB1618)	Phenylketonuria	NCT03516487	Phase 1/2a	Ongoing
Metabolic diseases	Bacteroides (NB1000S)	Enteric hyperoxaluria	NCT04909723	Phase 1/2a	Ongoing
Cancer	*B. longum* (APS001F)	Solid tumor	NCT01562626	Phase 1/2	Ongoing
Cancer	*B. animalis lactis* (EDP1503)	Colorectal cancer and checkpoint inhibitor relapsed tumors	NCT03775850	Phase 1/2	Done
Disease	*L. lactis* (AG011)	Moderately active ulcerative colitis	NCT00729872	Phase 1/2	Done
Metabolic diseases	*E. coli* (SYNB1020)	Cirrhosis and hyperammonemia	NCT03447730	Phase 1/2	Done
Metabolic diseases	*L. lactis* (AG019)	Type 1 diabetes	NCT03751007	Phase 1/2	Ongoing
Infection	*L. lactis* (AG013)	Oral mucositis	NCT03234465	Phase 2	Done
Infection	*L. crispatus* (LACTIN-V, Osel, Inc.)	Bacterial vaginosis	NCT02766023	Phase 2	Done
Infection	*L. crispatus* (LACTIN-V)	Urinary tract infection	NCT00305227	Phase 2	Done
Diseases	*L. rhamnosus GG* (LGG)	Gastroenteritis	NCT01773967	Phase 2/3	Done
Infection	*Salmonella* (Vivotif)	*S. typhi*	STN:103123	—	License
Infection	*Vibrio cholerae* (Vaxchora)	*V. cholerae* serogroup O1	STN:125597	—	License
Cancer	*Salmonella* (VNP20009)	Cancer neoplasm, neoplasm, metastasis	NCT00004988	Phase 1	Done
Cancer	*Salmonella* (VNP20009)	Advanced solid tumors	NCT00006254	Phase 1	Done
Cancer	IL-2 expressing, attenuated *Salmonella typhimurium*	Unresectable hepatic metastasis	NCT01099631	Phase 1	Ongoing
Metabolic diseases	*E. coli* (SYNB1020)	Urea cycle disorder	NCT03179878	Phase 1	Done
Cancer	*E. coli bacterial minicell* (VAX014)	Urothelial carcinoma of the urinary bladder	NCT03854721	Phase 1	Ongoing
Cancer	*Clostridium novyi-*NTspores	Malignant neoplasm of breast or digestive organs, etc	NCT03435952	Phase 1	Ongoing
Cancer	*Clostridium novyi-*NTspores	Solid tumor malignancies	NCT01924689	Phase 1	Done
Cancer	*Salmonella* (VXM01)	Recurrent glioblastoma	NCT03750071	Phase 1/2	Ongoing
Cancer	*Listeria monocytogenes* (ADXS31-142 + Pembrolizumab)	Cancer prostate cancer	NCT02325557	Phase 1/2	Ongoing
Cancer	*Listeria monocytogenes* (CRS-207)	Pancreatic cancer	NCT03190265	Phase 2	Ongoing
Infection	*E. coli* (ACE527)	Diarrhea	NCT03179878	Phase 1/2	Done
Metabolic diseases	*Cutaneous microbiota* (STMC-103)	Atopic immunoglobulin e-mediated allergic disorder	NCT03819881	Phase 1b	Unknown
Cancer	*MRx0518* *+* *Pembrolizumab*	Non-small-cell lung cancer, renal cell carcinoma, melanoma	NCT03637803	Phase 1/2	Ongoing
Cancer	*VE800* *+* *Nivolumab*	Metastatic cancer	NCT04208958	Phase 1/2	Ongoing
Infection	*Oral full-spectrum microbiota (CP101)*	*C. difficile* infection recurrence	NCT03110133	Phase 2	Done
Metabolic diseases	Full spectrum microbiota (FSM)	Autism spectrum disorder, gastrointestinal disorder	NCT03408886	Phase 2	Ongoing
Disease	DS-01	Irritable bowel syndrome	NCT04598295	Phase 2	Unknown
Metabolic diseases	MaaT013	Gastrointestinal acute graft vs host disease	NCT03359980	Phase 2	Done
Infection	VE303	*C. difficile* infection recurrence	NCT03788434	Phase 2	Done
Infection	SER-109 (purified Firmicutes spores)	*C. difficile* infection	NCT03183141	Phase 3	Done
Infection	RBX2660	*C. difficile* infection	NCT03244644	Phase 3	Done

*Bifidobacterium animalis*: *B. animalis*; *Lactobacillus crispatus*: *L. crispatus*.

## Data Availability

All relevant data are included within the article.

## Abbreviations

GEMs: Genetically engineered microorganisms
LAB: Lactic acid bacteria
ZFNs: Zinc finger nucleases
TALEN: Transcription activator-like effector nucleases
CRISPR-Cas: Clustered regularly spaced short palindromic repeats-CRISPR associated
WHO: World Health Organization
*E. coli*: *Escherichia coli*
HVT: Herpesvirus of Turkey
sgRNA: Single guide RNA
DSBs: Double-strand breaks
NHEJ: Nonhomologous end joining
HDR: Homology-directed repair
Indel: Insertion-deletion
BRED: Bacteriophage recombineering of electroporated DNA
tracrRNA: Trans-activating crRNA
TAR: Transformation-associated recombination
YAC: Yeast artificial chromosomes
IBS: Irritable bowel syndrome
IBD: Inflammatory bowel disease
*E. faecalis*: *Enterococcus faecalis*
*L. monocytogenes*: *Listeria monocytogenes*
BBB: Blood-brain barrier
*S. typhimurium*: *Salmonella typhimurium*
*M. abscessus*: *Mycobacterium abscessus*
dspB: Dispersin B
dpoL1: Depolymerase
*C. difficile*: *Clostridium difficile*
AMPs: Antimicrobial peptides
*L. gasseri*: *Lactobacillus gasseri*
*L. lactis*: *Lactobacillus lactis*
*L. casei*: *Lactobacillus casei*
*L. paracasei*: *Lactobacillus paracasei*
*L. jensenii*: *Lactobacillus jensenii*
HIV: Human immunodeficiency virus
*B. breve*: *Bifidobacterium breve*
*B. longum*: *Bifidobacterium longum*
EcN: *E. coli* Nissle
*V.cholerae*: *Vibrio cholerae*
*P. aeruginosa*: *Pseudomonas aeruginosa*
ICC: Immunocytochemistry
*S. boulardii*: *Saccharomyces boulardii*
*C. difficile*: *Clostridium difficile*
*V. vulnificus*: *Vibrio vulnificus*
*Fla*B: Flagellin B
TLR5: Toll-like receptor 5
*L. lactis*: *Lactococcus lactis*
*L. reuteri*: *Lactobacillus reuteri*
*S. aureus*: *Staphylococcus aureus*
CAI-1: Cholera autoinducer-1
AIP-I: Autoinducer peptide I
QS: Quorum sensing
*B. pseudomallei*: *Burkholderia pseudomallei*
*E. faecium*: *Enterococcus faecium*
KGF-2: Keratinocyte growth factor-2
TFF: Trefoil factor
IL: Interleukin
*B. ovatus*: *Bacteroides ovatus*
*La. casei*: *Lactococcus casei*
hLF: Human lactoferrin
LcrV: Low-calcium response V
*Y. pseudotuberculosis*: *Yersinia pseudotuberculosis*
*A. baumannii*: *Acinetobacter baumannii*
*K. pneumonia*: *Klebsiella pneumoniae*
*M. thermotolerans*: *Marinactinospora thermotolerans*
*S. nodosus*: *Streptomyces nodosus*
*S. lividans*: *Streptomyces lividans*
*B. subtilis*: *Bacillus subtilis*
*B. licheniformis*: *Bacillus licheniformis*
BCG: Biosynthetic cluster genes
PKS: Polyketide synthases
NRPS: Nonribosomal peptide synthetases
GPAs: Glycopeptide antibiotics
*S. coelicolor*: *Streptomyces coelicolor*
GPAHex: Glycopeptide antibiotic heterologous expression system
*S. roseosporus*: *Streptomyces roseosporus*
MRSA: Methicillin-resistant *S. aureus*
SAM: S-Adenosyl methionine
HRDRs: Host-range-determining regions
DBTL: Design, build, test, and learn cycle
HTS: High-throughput screening
*S. typhimurium*: *Salmonella typhimurium*
LBPs: Live biotherapeutic products
IL-12: Interleukin-12
UC: Ulcerative colitis
CD: Crohn's disease
FMT: Fecal microbiota transplantation
*α*-MSH: *α*-Melanocyte-stimulating hormone
NO: Nitric oxide
*S. thermophilus*: *Streptococcus thermophilus*
ROS: Reactive oxygen species
TNFR1: Tumor necrosis factor-ɑ receptor
H-SN1: Hydrostatin-SN1
*H. cyanocinctus*: *Hydrophis cyanocinctus*
MAPK: Mitogen-activated protein kinase
IFN: Interferon
PBMCs: Peripheral blood mononuclear cells
*A. thaliana*: *Arabidopsis thaliana*
NAPEs: N-Acylphosphatidylethanolamines
NAE: N-Acylethanolamide
*K. aerogenes*: *Klebsiella aerogenes*
GLP-1: Glucagon-like peptide-1
PDX-1: Duodenal homeobox 1
PKU: Phenylketonuria
AIs: Autoinducers
AI-2: Autoinducer-2
CAI-1: Cholera autoinducer 1
*H. pylori*: *Helicobacter pylori*
VRE: Vancomycin-resistant *Enterococcus*
EHEC: Enterohemorrhagic *E. coli*
BACs: Bacterial artificial chromosomes.
